# Food Timing, Circadian Rhythm and Chrononutrition: A Systematic Review of Time-Restricted Eating’s Effects on Human Health

**DOI:** 10.3390/nu12123770

**Published:** 2020-12-08

**Authors:** Réda Adafer, Wassil Messaadi, Mériem Meddahi, Alexia Patey, Abdelmalik Haderbache, Sabine Bayen, Nassir Messaadi

**Affiliations:** Department of General Medicine, Henri Warembourg Faculty of Medicine, University of Lille, 59000 Lille, France; wassilme@gmail.com (W.M.); meddahi_meriem@hotmail.fr (M.M.); alexia.patey@gmail.com (A.P.); abdelmalik.hader@gmail.com (A.H.)

**Keywords:** time-restricted feeding, time-restricted eating, intermittent fasting, circadian rhythm, systematic review

## Abstract

Introduction: Recent observations have shown that lengthening the daily eating period may contribute to the onset of chronic diseases. Time-restricted eating (TRE) is a diet that especially limits this daily food window. It could represent a dietary approach that is likely to improve health markers. The aim of this study was to review how time-restricted eating affects human health. Method: Five general databases and six nutrition journals were screened to identify all studies published between January 2014 and September 2020 evaluating the effects of TRE on human populations. Results: Among 494 articles collected, 23 were finally included for analysis. The overall adherence rate to TRE was 80%, with a 20% unintentional reduction in caloric intake. TRE induced an average weight loss of 3% and a loss of fat mass. This fat loss was also observed without any caloric restriction. Interestingly, TRE produced beneficial metabolic effects independently of weight loss, suggesting an intrinsic effect based on the realignment of feeding and the circadian clock. Conclusions: TRE is a simple and well-tolerated diet that generates many beneficial health effects based on chrononutrition principles. More rigorous studies are needed, however, to confirm those effects, to understand their mechanisms and to assess their applicability to human health.

## 1. Introduction

Eating behaviors are the most influential factor in the development of chronic pathologies [1,2]. The main food risk factors identified are the excessive consumption of salt, sugars, processed meats, soda drinks and an insufficient intake of fruits, vegetables, cereals and polyunsaturated fats [3,4].

Data on eating behaviors generally evaluate the qualitative and quantitative aspects of nutrition. However, little information exists on the temporal characteristics of food and their impact on the occurrence of diseases.

The daily feeding time is the period from the start of the first meal to the end of the last meal of the day. An American cohort of 15,000 adults estimated this feeding time to be 12 h for most individuals, and it even reached 15 h for more than half of them [5]. An Indian study found the same results and suggested that the lengthening of the daily feeding time may be a factor in the development of metabolic disorders [6].

Thus, recent observations have indicated that reducing the daily feeding time may limit the development of noncommunicable diseases. For example, Marinac et al. suggest that prolonging the length of the nightly fasting interval may be a strategy for reducing the risk of breast cancer recurrence [7]. A cohort of 420 individuals showed that eating late may negatively influence weight loss [8] and the authors of a study on a cohort of 2650 adult women suggested that reducing evening energy intake and fasting for longer nightly intervals may lower systemic inflammation and subsequently reduce the risk of breast cancer and other inflammatory and metabolic diseases [9].

Intermittent fasting (IF) is a dietary intervention that alternates between a period of fasting and a period of normal eating (1–3 days per week) [10,11]. Among various forms of IF, alternate day fasting (ADF) is defined as a continuous sequence of a fast day (i.e., 100% energy restriction) followed by and a feed day (ad libitum food consumption), resulting in 36-h fasting [12,13]. IF represents a dietary approach of growing interest as a weight loss and health improvement strategy [14,15,16].

Time-restricted feeding (TRF) is a form of IF that limits the daily food consumption to a period of 4–12 h, which induces a fasting window of 12–20 h per day [17]. However, TRF differs from IF in two aspects: (1) TRF does not require caloric restriction and (2) it requires a consistent daily eating window [18].

This dietary approach has shown beneficial effects in animals. In rodent models, it has been demonstrated that TRF protects mice fed a high fat diet against obesity, hyperinsulinemia, hepatic steatosis and inflammation [19]. Another study showed that TRF attenuates the onset of metabolic diseases and reverses the progression of metabolic diseases in mice with pre-existing obesity and type II diabetes, hepatic steatosis and hypercholesterolemia [20]. A study on *Drosophila* showed that TRF attenuates age-related cardiac decline [21].

The time-restricted eating (TRE) is used to refer to human models. Human trials and systematic reviews on TRE have supported the findings of animal studies and have demonstrated beneficial effects on cardiovascular metabolism [22]. A recent systematic review and meta-analysis of 19 studies showed that TRE leads to weight loss and a reduction in fat mass with a preservation of fat-free mass and also has beneficials effects on cardiometabolic parameters such as blood pressure, fasting glucose concentration and cholesterol profiles [18].

The purpose of this study is to systematically review the international literature, exploring the effects of TRE on human health.

## 2. Method

This work is a systematic descriptive review of the literature, following the international PRISMA recommendations [23].

### 2.1. Collection and Selection of Items

The collection and selection of articles was carried out by three independent researchers from September 2019 to September 2020 using the keys words time-restricted feeding, time-restricted eating and time-restricted nutrition.

The screened databases were MEDLINE, Web of Science, Scopus, Science Direct and the Cochrane Library. In addition, the following specialized journals were analyzed: *Nutrition Reviews* from the Oxford Academy, *Nutrition Reviews* from the Wiley Online Library, *Obesity* from the Wiley Online Library, the *American Journal of Clinical Nutrition*, *Nutrition—Annual Review of Nutrition* and *Clinical Nutrition*. The research equations used for each database are listed in Supplemental Method S1.

The included articles were all clinical trials, published between January 2014 and September 2020, in English, evaluating TRE on adult human populations.

Literature reviews, meta-analyses and any other type of publication were excluded, as were studies on Muslim fasting.

Once the articles were collected, they were selected through a process including three steps. The first was the selection of articles based on a reading of their titles. The remaining articles were then selected based on their abstract. Finally, the full article was read completely before being included or excluded according to the abovementioned criteria.

All the selection steps were carried out blindly by three operators. In the case of disagreement, a discussion between the three operators and the supervisor was carried out until consensus was reached.

For the constitution of the database, the authors used the Rayyan QCRI web application, specialized in systematic literature reviews [24].

### 2.2. Data Analysis and Extraction

The data were extracted from the original articles and classified in a table (Supplemental Table S1) according to the following themes: reference of the article, type of study, characteristics of the participants, main outcomes, main results and level of evidence. These data were then synthesized in a table for analysis. Data extraction and analysis was carried out by a single operator, the author of the review.

All the included articles will be referred to in this paper as RXX, with XX being a number.

### 2.3. Level of Evidence Classification

Levels of evidence were classified according to the Downs and Black checklist [25], which is a 27-item validity score assessing all the methodological aspects of clinical trials. Two questions about the double-blind method (14 and 15) were uncounted, which brought the score to 25 (Supplemental Table S2).

The second indicator was the classification provided by the High French Health Authority (HAS) [26] (Supplemental Table S3—HAS gradation).

The combination of these two indicators allowed for the determination of three levels of evidence—high, medium and low.

## 3. Results and Discussion

### 3.1. Selected Articles and Characteristics

**Figure 1:**
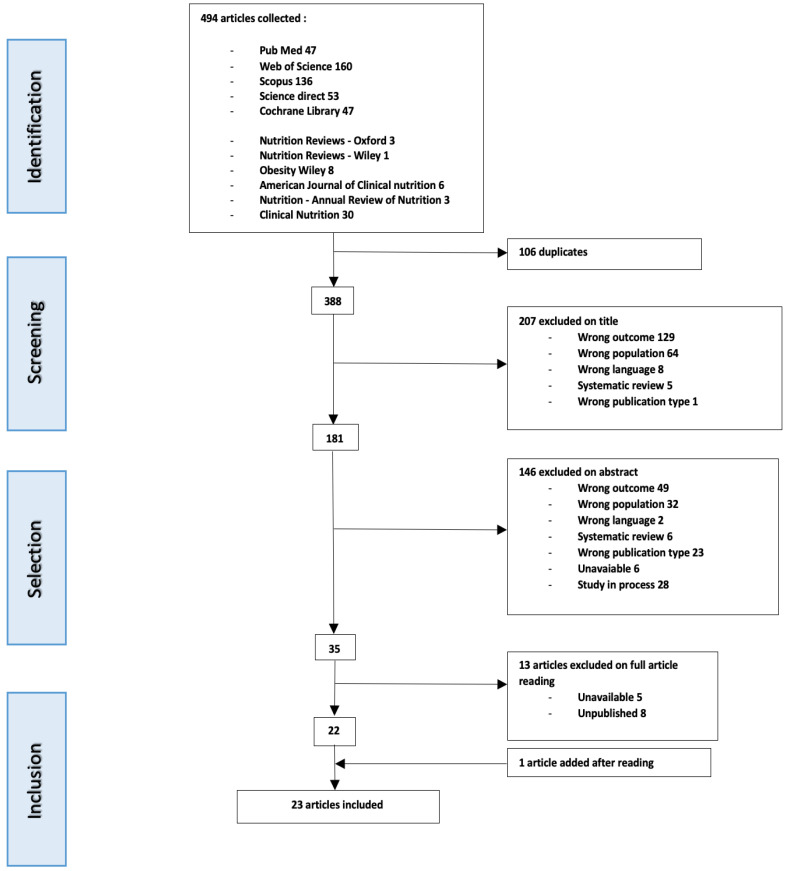
Flow chart of article selection strategy.

Preliminary research identified four hundred and ninety-four articles, including clinical trials, literature reviews and other relevant work. After the exclusion of one hundred and six duplicates, three hundred and eighty-eight articles remained, from which 22 have been finally selected [27,28,29,30,31,32,33,34,35,36,37,38,39,40,41,42,43,44,45,46,47,48,49]. An article was added during the full article reading stage (R15—[41]), making a total of 23 articles included for analysis (see flow chart in Figure 1).

The literature on TRE is limited, with only 23 articles having been published, reporting the results of 22 trials. The studies took place over periods ranging from four days to four months and involved groups of 8 participants for the smallest sample and 105 participants for the largest one. The overall level of evidence was low to medium, with 10 controlled randomized trials with short intervention periods (average of 33.5 days) and small samples (average *n* = 21) for the medium-quality evidence studies. The other 11 studies were of low quality, with three non-randomized controlled studies and eight single-arm non-controlled trials. One study had a high level of evidence, with a controlled and randomized protocol and the largest sample of the review trials (105 participants) (R23).

However, it is important to note that 28 experiments evaluating TRE were still in progress at the closure of the collection period (March 2020). Among these 28 trials, 26 are randomized and involve larger samples (mean *n* = 81), which will provide better quality evidence on the subject. These observations indicate that TRE is a research subject of growing interest and knowledge.

### 3.2. Types of Time-Restricted Eating: Clarification of Terms

Time-restricted feeding is an intermittent fast which consists in limiting the daily feeding period [17]. However, the definition of the restriction is still imprecise in studies on humans and varies from 8 to 12 h, depending on the sources.

First, the nomenclature used to describe the type of time-restricted feeding practiced is standardized: TRE followed by the number of hours of feeding and fasting in a 24-h period, separated by a slash. For example, TRE 8/16 indicates a restriction of the daily feeding period to 8 h for a 16 h fast.

Among the 22 studies in the review, only one practiced a restriction equal to 12 h or less (R13), all the others having limited daily nutrition to a window of 10 h or less. These data clarify the definition of TRE in humans and indicate that it starts from a period of 10 h, a daily fast of 14 h and more.

The 8/16 variant is the most common form of TRE, with 50% of the trials in the review (*n* = 12). One TRE protocol (R1) did not impose any hours of fasting but required participants to shift their first meal and advance the time of their last daily meal by an hour and a half.

The food window can also vary during the day. For example, Sutton et al. (R4) introduced the concept of early TRE (eTRE), in which the feeding period started earlier in the day (R2, R4 and R5). By extension, delayed or late TRE (dTRE) delays the intake of the first meal (R3, R7, R8, R9, R12, R14 and R23). Hutchinson et al. (R11) have evaluated both early and late TRE. Three studies (R16, R17 and R18) allowed the self-selection of the food intake period by the participants.

TRE is generally an every-day (except for R12 and R13), water (except for R13) and ad libitum fast, which means that participants can eat until satiety without any restriction on the quality of quantity of food intake, except for four studies which used iso-caloric protocols (R2, R4, R19, R21).

These characteristics show that TRE could be adaptable to the participants’ eating habits, which may positively influence dietary adherence.

### 3.3. Adherence to TRE and Effect on Calorie Consumption

TRE was overall well-tolerated, with an adherence rate superior to 80% in eight studies (R6, R10, R12, R16, R17, R18, R20, R21) on the 10 that have evaluated adherence (Table 1). However, Gabel et al. (R7) have reported a dropout rate of 24% and in another feasibility study (R22), the compliance to the 9/15 TRE was 72 ± 24% (i.e., ~5 days/week).

Otherwise, adherence to meal timing was self-declared by the participants in diet diaries or interviews for most of the studies (R1, R3, R6, R7, R10, R11, R12, R13, R14, R16, R17, R19, R22, R23). Food intake measurement was also based on participants’ self-declarations (R1, R7, R8, R9, R12, R13, R14, R19, R22, R23). The food intake was not even tracked in seven of the studies (R3, R6, R10, R11, R16, R17, R20). These limitations could lead to declaration and confusion bias.

This limitation is an inherent difficulty in measuring health behaviors like food intake in free-living conditions. Gill and Panda (R15) developed a smartphone application which combines a photograph and an optional notification added by the participant (myCircadianClock, mCC—www.mycircadianclock.org). This method, also used in Wilkinson et al. (R18) and Chow et al. (R20), can represent an interesting way to reduce the methodological bias involved in food tracking. Studies have thereby shown that using a mobile application is more accurate for monitoring compliance to a dietary protocol than a traditional diet diary, and that it can also improve adherence to this protocol [50].

A recent literature review indicated that the main determinants of adherence to a diet are the ability to reduce the desire to eat and to conform to the patient’s eating habits [51]. In the present review, hunger remained stable in four studies (R4, R5, R11, R21), and in Ravussin et al. (R5) participants reported an improvement in several components of appetite by reducing hunger discomfort and the desire to eat. In addition, time-restricted eating appears less restrictive than classic diets, with no limitation in the quality or quantity of the food, and thus more adaptability to the participants’ eating behaviors.

Not only the adherence, but the long-term adherence is the most important factor of the success of a diet on overall health benefits [52]. The studies in this review were four days to 4 months long, which is a relatively short time to conclude. In the trial conducted by Wilkinson et al. (R18), 63% of the participants were somehow engaged in TRE 16 months after the end of the 12-week intervention. This result suggests that TRE could be a sustainable dietary intervention and it would be interesting to evaluate adherence over a longer period (12 to 24 months).

One may have expected that the restriction of feeding time would increase the calorie intake. Inversely, TRE reduced caloric consumption by 20% on average, without an alteration of macronutrient distribution (Table 1). Interestingly, this calorie restriction has been described as not intentional by the participants, which is likely to protect them against cognitive restriction. A study revealed that cognitively-restrained eaters increase their energy intake compared to baseline and lose less body weight than unrestrained eaters after a 10-month dietary intervention [53].

Moreover, TRE provides a similar level of calorie restriction to other voluntary restrictive dietary measures, such as continuous caloric restriction. Indeed, the CALERIE is the largest ever study to evaluate continuous caloric restriction in a non-obese population [54] and its results indicated that even the most motivated subjects were unable to maintain a 25% calorie restriction over two years. Put together, these findings suggest that (1) TRE could represent a more sustainable strategy for patients who want to reduce their caloric intake and (2) TRE could produce a more sustainable body weight loss than voluntarily restrictive diets.

### 3.4. Metabolic Effects of TRE

It has been established in the literature that intermittent fasting produces beneficial metabolic effects via caloric restriction [55,56]. This principle is confirmed by the results of the review, with an average weight loss of 3% and fat mass perdition (Table 1), body fat loss and a decrease in the level of fasting glucose concentration (R1), weight loss and BMI reduction in (R7, R9, R15, R18), a drop in systolic blood pressure (R7, R9, R18), a reduction in waist circumference and a decrease in cholesterol concentration (R18).

Interestingly, six studies on TRE have shown beneficial metabolic effects, regardless of the calorie restriction (Table 1). For example, a randomized iso-caloric study evaluating eTRE showed a decrease in the average blood sugar level and reduced insulin resistance (R2). Likewise, a crossover randomized trial (R21) demonstrated that short-term TRE improved nocturnal glycemic control. An iso-caloric trial on eTRE 8/16 (R4) showed an improvement in glucose tolerance and a major decrease in systolic blood pressure, comparable to the results obtained with pharmacological treatment with an ACE inhibitor [57]. Moro et al. reported that a combination of TRE 8/16 with regular physical activity reduced fat mass, decreased blood glucose levels, improved insulin resistance and lowered triglyceride levels (R8). Grant et al. (R14) showed a reduction of 4%–7% of fat mass, despite an increase in caloric intake between groups, but only on a per protocol analysis. McAllister et al. showed body weight and fat mass loss, a reduction in systolic blood pressure and an increase in HDL cholesterol in two groups applying 8/16 TRE in ad libitum and isocaloric conditions, with no difference between groups (R19). The authors suggested that this effect was due to the increase in adiponectin levels. Adiponectin is an adipokine of which the secretion increases during fasting [58] and which could produce a loss of fat mass by increasing energy expenditure [59,60] and by activating AMPK, a kinase which stimulates lipolysis and insulin-sensitivity [61,62,63]. An adiponectin level increase was also observed in the randomized study by Moro et al. (R8). These findings are interesting and suggest that the TRE could produce positive metabolic effects independently of energy balance.

Although metabolic changes are the result of an imbalance between the energy content of food eaten and energy expended partly via physical activity [64,65], only three of the six mentioned studies measured physical activity (R8, R14, R21). In the Moro et al. trial, for example (R8), 8-week TRE associated with resistance training (RT) produce the abovementioned metabolic effects in comparison with a group of adults who performed only strength training, with no difference in food intake and physical activity. In this study, the physical activity was only measured during the training session, thus the stated effects could be due to an unmeasured energy expenditure increase outside of training sessions. Tinsley et al. also evaluated the effects of TRE associated with resistance training (R4). They measured physical activity during and outside training sessions, which limits the potential bias. It is necessary for further studies on TRE to systematically control the impact of physical activity to confirm these interesting effects.

### 3.5. TRE and the Circadian Clock

It has also been established that the beneficial metabolic effects of a diet are induced by the weight and/or fat loss it produces [56]. This assertion has been confirmed in seven studies of this review (R1, R3, R7–9, R8, R17, R18, R19—Table 1). Wilkinson et al., for example, performed a 12-week 10/14 TRE on 19 adults with metabolic syndrome, and recorded LDL and non-HDL cholesterol reduction, a decrease in systolic blood pressure associated with a body weight loss of 3%, body fat perdition and the reduction in BMI and waist circumference. In contrast to other diets, TRE generates metabolic benefits regardless of any loss of weight or fat. For example, Hutchinson et al. showed an improvement in the glucose tolerance and a decrease in triglycerides in participants practicing eTRE for two weeks (R11). Furthermore, Sutton et al. recorded an improvement in glucose-tolerance and a decrease in insulin levels and blood pressure (R4). These promising results suggest that TRE generates an intrinsic metabolic effect, independently of weight loss or fat mass.

The main mechanism hypothesized to explain this intrinsic effect is its action on the body’s circadian rhythm [66]. The circadian system represents all the physiological processes involved in a 24-h cycle, such as the sleep/wake cycle, blood pressure, heart rate, hormone secretion, cognitive performance and mood regulation [66]. The system is regulated by a number of environmental stimuli such as food intake, light exposure and physical activity [67]. Growing evidence shows that the disruption of the circadian system is the cause of many metabolic pathologies like obesity [68,69]. Thus, limiting the time of food consumption seems to readjust the food intake with the circadian clock [70,71,72,73]; this is a fundamental principle of chrononutrition, which is the study of relationships between circadian clocks and food intake [74] and which suggests that meal timing affects metabolism [75].

Several results support this hypothesis. First, adiponectin has been established to play a key role in all the metabolic effects described above, such as the loss of fat mass [62], promotion of glycemic and lipid metabolism [60,63] and reduction in blood pressure [76]. This molecule is known to actively regulate the action of the circadian system [77,78]. In addition, a randomized controlled iso-caloric study involving large-scale gene expression analysis showed that eTRE affected the expression of six genes involved in circadian rhythm (R2). Finally, a study indicated that TRE produced a significant and durable improvement of sleep quality (R15) and a 12-week single-arm trial evaluating 10/14 TRE on 19 adults recorded an increase in sleep duration and efficiency in 84% of the participants. Moreover, the improvement of sleep quality is known to be directly linked to the enhancement of the circadian system [79,80]. However, the exact action of TRE on circadian systems remains unclear in humans, and studies are needed to better understand the mechanisms involved.

A growing number of observations on animal models hypothesize that the positive effects of physical activity on cardiovascular markers are also due to the ability to restore the circadian rhythm [81,82]. Moro et al. (R8) compared a group combining a TRE diet and an RT program (three sessions/week) with a group practicing RT alone for eight weeks. Only the TRE + RT group recorded a decrease in total body mass and fat mass as well as an improvement in metabolic markers (glycemic tolerance, decrease in triglycerides). However, as mentioned above, energy expenditure through physical activity was only recorded during training sessions, which could lead to a classification bias. Future studies should systematically control this factor and could test TRE alone vs. TRE + RT. Again, smartphone-based methods represent an interesting strategy to measure physical activity and energy expenditure in normal living conditions [83].

### 3.6. Other Effects of TRE

Other beneficial effects were explored in our review. Intermittent fasting has been shown to prevent cancer by lowering the IGF-1 level in animals [84] and humans [84]. This effect has been stated in two review studies (R2 and R8) and suggests a protective effect of TRE in carcinogenesis. In addition, researchers have shown that an intermittent one-month fast leads to a decrease in pro-inflammatory cytokines [85]. This result was also confirmed by a review study which reported a drop in TNF-α and IL-1-β levels after 8 weeks of TRE associated with physical training (R8). The decrease in these mediators of inflammation is otherwise hypothesized to play a role in circadian rhythm improvement through the action of adiponectin [86]. Gene expression analysis has shown that TRE increases the expression of four genes involved in autophagy and longevity (R2). This study was also the first to show that TRE increases the secretion of BDNF, a protective factor against the development of neurodegenerative diseases [87]. In addition, two studies exploring the effects of TRE in the elderly showed an improvement in walking speed, a predictor of geriatric robustness [88], as well as an attenuation of immuno-senescence process (R10 and R13). Furthermore, Sutton et al. (R4) showed that TRE causes a significant decrease in 8-isoprostane, a biomarker of oxidative stress [89]. Although they are promising, these observations are based on biological criteria whose clinical implications are limited. Longitudinal studies are needed to assess the impact of TRE on the morbidity and mortality linked to these.

## 4. Conclusions

Our review revealed that reducing the daily feeding period is a well-tolerated dietary approach for calorie restriction, which would be interesting to evaluate on longer term. Interestingly, TRE differs from other dietary interventions by producing beneficial effects on many health markers regardless of the energy balance. These effects suggest that nutrition affects health not only through quantity or quality of the intakes, but also via the timing of food consumption according to the circadian clock.

More widely, it would be interesting to evaluate whether other health behaviors like sleep quality or physical activity can influence health markers by their timing according to the circadian clock. For this purpose, further studies could use smartphone-based methods to measure health behaviors in free-living conditions. Indeed, smartphones are ubiquitous and present worldwide, available at any time of the day and can be used as portable monitoring devices to provide detailed information on lifestyle behaviors according to the circadian clock. This information should help to better understand the relationship between health behaviors, timing and the occurrence of diseases.

## Figures and Tables

**Table 1:** Synthesis of results.

Study [Ref]	Level of Evidence	Population	Protocol	Energy Balance	Metabolic Effect	Other Results
R1 Antoni et al. 2018 [27]	Low	*n* = 13 Healthy adults, 29–57 years.	↓ Food window of 3 h 10-week non-randomized controlled trial.	↓ daily energy intake	↓ body 1.9% fat mass index ↓ fasting blood glucose	Only 19% withdrawal including one lost to follow-up
R2 Jamshed et al. 2019 [28]	Medium	*n* = 11 Healthy adults, 32 ± 7 years.	Early TRE 6/18 4-day randomized controlled iso-caloric crossover trial with 3.5 to 5 weeks of wash-out	No difference in calorie intake (iso-caloric)	↓ 24-h glucose and hyperglycemic excursion ↓ insulin resistance ↑ total cholesterol, LDLc, HDLc	↑ BDNF ↓ IGF1 Modification of genes expressions involved in circadian rhythm, longevity, autophagy
R3 Smith et al. 2017 [29]	Low	*n* = 20 Healthy women, 21.3 ± 1.2 years.	Delayed TRE 8/16 4-week single-arm trial	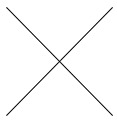	↓ body mass of 0.6 ± 1 kg ↓ body fat in participants that strength trained (>3 day/week)	
R4 Sutton et al. 2018 [30]	Medium	*n* = 8 Pre-diabetic overweighted men, 59 ± 9 years.	Early TRE 6/18 5-week controlled, randomized, isocaloric crossover trial with 7 weeks of wash-out	No difference in calorie intake (iso-caloric)	↓ insulin (fasting, mean and peak) ↑ insulin sensitivity ↓ insulin resistance ↑ triglycerides ↓ blood pressure	↓ desire to eat ↓ 8-isoprostane
R5 Ravussin et al. 2019 [31]	Medium	*n* = 11 Healthy adults, 32 years.	Early TRE 6/18 4-day controlled, randomized, iso-caloric crossover trial with 3.5 to 5 weeks of wash-out	No difference in calorie intake (standardized meals) Energy expenditure unchanged	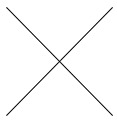	↓ several aspects of hunger ↓ in morning ghrelin, leptin and GLP-1 ↓ average ghrelin ↑ in evening PYY (satiety)
R6 Gabel et al. 2019 [32]	Low	*n* = 23 Obese, 50 ± 2 years.	TRE 8/16 12-week single-arm trial.	Physical activity unchanged. No measure of calorie intake	↓ 4% weight ↓ 5% fat mass	80% mean adherence.
R7 and 9 Gabel et al. 2018, 2014 [33,34]	Low	*n* = 23 Obese, 50 ± 2 years.	Delayed TRE 8/16 12-week, non-randomized controlled trial with matched historical group.	↓ of 350 kcal/day Physical activity unchanged.	↓ 2.6% of relative weight ↓ of relative BMI ↓ systolic blood pressure of 7 ± 2 mmHg	74% adherence rate. No one in the TRE group reported dropping out due to issues with the diet.
R8 Moro et al. 2016 [35]	Medium	*n* = 34 Adults who strength train, 29.21 ± 3.8 years.	TRE 8/16 + RT 8-week randomized controlled trial. TRE + RT vs. RT	No difference in calorie intake between groups No difference in physical activity during training sessions	↓ fat mass ↓ blood glucose levels ↓ insulin resistance ↓ triglycerides	↓ TNF-α ↓ IL-1 β ↓ IGF 1 ↑ adiponectin ↓ respiratory ratio (lipid oxidation) Conservation of muscular mass and strength
R10 Anton et al. 2019 [36]	Low	*n* = 10 Overweighted elderly adults, 77.1 years.	TRE 8/16 4-week single-arm trial.	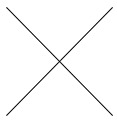	Mean weight ↓ of 2.6 kg	↑ walking speed Improvement in mental and physical function. 84% mean adherence.
R11 Hutchison et al. 2019 [37]	Medium	*n* = 15 Pre-diabetic men, 55 ± 3 years.	dTRE 9/15 vs. eTRE 1-week cross-over, randomized trial with 2 weeks of wash-out	No difference in physical activity No measure of calorie intake	↓ glucose AUC and mean fasting glucose in eTRE ↓ triglycerides in two groups	No effect of TRF on perceived hunger, fullness, or desire to eat.
R12 Tinsley et al. 2016 [38]	Medium	*n* = 18 Adults who strength-train, 22 ± 2.4 years.	TRE 4/20 + RT vs. RT alone. 8-week randomized controlled trial.	↓ of 650 kcal/day between fasting days and non-fasting days ↓ weekly calorie intake	No significant change in weight and fat mass	Conservation of lean mass, muscular volume and muscular strength. 95% mean adherence.
R13 Gasmi et al. 2017 [39]	Medium	*n* = 40 20 y (*n* = 40) vs. 50 years (*n* = 20)	TRE 12/12: TRE 50 years + 20 years Control 50 years + 20 years 12-week randomized, controlled trial.	No difference in calorie intake.	No change in body composition and muscular function	↓ immuno-senescence
R14 Tinsley et al. 2019 [40]	Medium	*n* = 40 Women who strength-train 18–30 years.	Delayed TRE 8/16 8-week randomized controlled trial. -RT + placebo -TRE + RT + placebo -TRE + RT + HMB	↑ calorie intake from 20 to 200 kcal/day No difference in physical activity and REE	↓ fat mass of 4%–7% in per protocol analysis for the 2 TRE groups	No side effects in 90% of participants at the end of the protocol
R15 Gill et al. 2015 [41]	Low	*n*= 8 Obese adults, 18 years.	TRE 10/14 every day, 3-week single-arm trial. Smartphone-based assessment of caloric quantity and timing intake	↓ calorie intake of 20%	↓ weight by 4% ↓ BMI by 1.15 kg/m^2^	↑ sleep quality ↓ hunger
R16 Lee et al. 2020 [42]	Low	*n* = 10 Overweight sedentary elderly adults, 77.1 years.	TRE 8/16 every day with self-selection of eating window. 4-week single-arm trial.	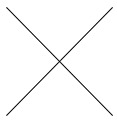	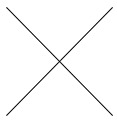	Mean adherence of 84%.
R17 Kesztyüs et al. 2019 [43]	Low	*n* = 40 Abdominally obese, 49.1 ± 12.4 years.	TRE 8/16 every day with self-selection of the food intake period 12-week single-arm trial.	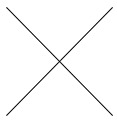	↓ weight of 1.7 ± 2.5 kg ↓ BMI of 0.6 ± 0.9 kg/m^2^ ↓ WC −5.3 ± 3.2 cm ↓ HbAc1 by 1.4 ± 3.5 mmol/mol	Mean adherence of 86 ± 15%
R18 Wilkinson et al. 2020 [44]	Low	*n* = 19 Adults with MetS 59 ± 11 years.	TRE 10/14 every day with self-selection of the food intake period. 12-week single-arm trial.	↓ by 8.62% ± 14.47%. No difference in physical activity.	↓ body weight (−3%) ↓ BMI (−3%) ↓ body fat (−3%) ↓ visceral fat rating (−3%) ↓ WC-4.46 ± 6.72 cm ↓ total cholesterol ↓ LDLc, ↓ non-HDLc ↓ systolic and diastolic BP	Mean adherence of 85 ± 12%. 63.2% participants were somehow engaged in TRE at 16 ± 4 months. ↑ in sleep duration by 12.45 min. ↑ in sleep duration and efficiency in 84% of participants.
R19 McAllister et al. 2019 [45]	Medium	*n* = 22 Physically active men, 22 ± 2.5 years.	TRE 8/16 every day ad libitum vs. Isocaloric (↓ 300 kcal from baseline). 4-week randomized controlled trial.	No difference in calorie intake	↓ body mass in both groups ↓ body fat mass in both groups ↓ systolic BP in both groups ↑ HDLc in both groups	↑ adiponectin in both groups. Improvement in subjective outcomes (alertness, energy, focus, mood) in ad libitum.
R20 Chow et al. 2020 [46]	Low	*n* = 20 Overweight adults with prolonged eating window (15.4 ± 0.9 h/day). 45.5 ± 12 years.	TRE 8/16 ad libitum every day. 12-week controlled non-randomized trial. TRE 8/16 group vs. non-TRE group.	No difference in physical activity. No measure of calorie intake.	(1) vs. non-TRE group ↓ body weight ↓ lean mass ↓ visceral fat (2) vs. preintervention measures ↓ body weight ↓ fat mass ↓ lean mass ↓ visceral fat	↓ of eating window in TRE group (9.9 ± 2 h) compared with non-TRE group. Adherence in TRE: 83.1% Correlation between restriction of eating window with fat and visceral masses loss
R21 Parr et al. 2020 [47]	Medium	*n* = 11 Overweight/obese and sedentary men. 38 ± 5 years.	TRE 8/16 every day vs. non-TRE (15 h/day). 5-day randomized crossover trial with a 10-day wash out period.	No difference in calorie intake (iso-caloric). No difference in physical activity.	↓ nocturnal glucose AUC TRE group ↓ peak insulin concentrations at breakfast in TRE group ↓ peak glucose concentration at breakfast in TRE group	100% adherence. Improvement of subjective feelings (well-being and satisfaction) ↓ evening hunger in TRE group
R22 Parr et al. 2020 [48]	Low	*n* = 19 Obese adults with T2D. 50 ± 9 years.	TRE 9/15 every day. 4-week singe-arm non-randomized trial	No difference in calorie intake. Adherence to TRE reduces calorie intake.	NS.	Mean compliance of 72 ± 24% (≅5 days/week).
R23 Miguet et al. 2020 [49]	High	*n* = 105 Overweight and obese adults. 46.5 ± 10.5 years.	dTRE 8/16 every day. 12-week controlled randomized trial. TRE 8/16 vs. control group.	No difference in calorie intake. No measure of physical activity.	↓ body weight in TRE group (1.17%) compared to baseline that was not significantly different from control group (0.75%).	

☒: No measurement. NS: non-significant modification (*p* > 0.05). Abbreviations: AUC: area under the curve; BDNF: brain-derived neurotrophic factor; BP: blood pressure; HDLc: HDL cholesterol; HMB: hydroxy-methyl-butyrate supplementation; IL: interleukin; insulin R: insulin resistance; insulin-S: insulin sensitivity; LDLc: LDL cholesterol; MetS: metabolic syndrome; REE: resting energy expenditure; RT: resistance training; T2D: type 2 diabetes; WC: waist circumference.

## Supplementary Materials

The following are available online at https://www.mdpi.com/2072-6643/12/12/3770/s1, Table S1: Data extraction, Table S2: Validity score provided by the Downs and Black checklist, Table S3: HAS gradation, Method S1: Research equations.
