# Supplementary material for: Food Timing, Circadian Rhythm and Chrononutrition: A Systematic Review of Time-Restricted Eating’s Effects on Human Health
Source: Nutrients. 2020 Dec 8;12(12):3770. doi: 10.3390/nu12123770 (PMC7763532; doi:10.3390/nu12123770)
Supplement: Supplementary file 1 [file nutrients-12-03770-s001.pdf]

FOOD TIMING, CIRCADIAN RHYTHM AND CHRONONUTRITION

A SYSTEMATIC REVIEW OF TIME-RESTRICTED EATING'S EFFECTS ON HUMAN HEALTH

SUPPLEMENTAL DATA

Supplemental Table 1 - Data extraction tables

A pilot feasibility study exploring the effects of a moderate time-restricted feeding intervention on energy intake, adiposity and metabolic physiology in free-living human subjects

Rona Antoni, Tracey M. Robertson, M. Denise Robertson and Jonathan D. Johnston

Journal of Nutritional Science (2018), vol. 7, e22, page 1 à 6 - Published the 6th July 2018

	**Characteristics of the population**	**Study design**	**Main outcomes**	**Main results**	**Strength and limitations**	**Level of evidence**
**R1**	13 healthy adults.<br><br>Target age : 29-57 y.	10-week non-randomized controlled trial.<br><br>Guildfort, United Kingdom.<br><br>**Protocol**<br>Delay the intake of the first meal and advance that of the last meal by 1.5 hours (total time restriction of 3 hours) accordind to eating time habits, without restriction in the frequency or quality of meals.	Adherence to the protocol by a guided diet diaries including :<br>-feeding time<br>-feeding intake<br>-open questionnaire on compliance and appetite.<br><br>Anthropometric and body composition by bio impedance.<br><br>Biomarkers of glycemic and lipid metabolism.	**Adherence**<br>↓ feeding time of 4 hours per day on average ($p < 0.001$) with a change from 12.4 to 8.6 hours / day loss of 3 participants out of 16, only one of whom was lost to follow-up.<br><br>↓ daily energy intake ($p = 0.0019$):<br>-3 participants reported an increase in the consumption of prepared meals due to the time constraint to prepare meals.<br>-5 participants consumed less alcohol<br>-No significant disturbance in the distribution of macronutrients.<br><br>57% of participants (n = 4) noted that the caloric reduction was linked to the reduction in appetite and eating opportunities and / or the reduction in snacking (especially in the evening).<br><br>**Body composition and biological measurements**<br>↓ 1.9% fat mass index ($p = 0.047$) and fasting blood sugar ($p = 0.008$).	**Limitation**<br>Non-randomized study<br><br>No precise defintion of the adherence<br><br>Small sample and composed almost exclusively of women (12/13): limits the generalizabiliy of the results<br><br>No objective measurement of physical activity<br><br>Subjective measurement of food intake and timing<br><br>Bio-impedance is a validated technique for measuring body composition but known to underestimate the rate of fat mass.	HAS : 2.<br><br>Validity score : 17/25.

Early Time-Restricted Feeding Improves 24-Hour Glucose Levels and Affects Markers of the Circadian Clock, Aging, and Autophagy in Humans<br>Humaira Jamshed, Robbie A. Beyl, Deborah L. Della Manna, Eddy S. Yang, Eric Ravussin and Courtney M. Peterson<br>*Nutrients*. 2019 Jun ; 11(6): 1234 - Published the 30th May 2019.						
	**Characteristics of the population**	**Study design**	**Main outcomes**	**Main results**	**Strength and limitations**	**Level of evidence**
**R2**	11 healthy adults.<br><br>Mean age : 32 years ± 7 years.	Randomized controlled iso-caloric cross-over trial of 4 days with a wash period of 3.5 to 5 weeks.<br><br>Alabama, United States of America.<br><br>**Protocol**<br>Early TRF 6/18 (meal 8.00 a.m, 11.00 a.m and 2.00 p.m) vs. Control arm (meal at 8.00 a.m 2.00 and 8.00 p.m)<br><br>Free meals on day 1 and 2<br><br>Standardized meals on day 3 and 4	Measurement of energy expenditure in a calorimetric chamber for 24 hours on day 4.<br><br>Assay of metabolic biomarkers :<br>-glycemic with 24 hr continuous glucose monitorage (CGM) and calculation of the HOMA-IR index reflecting insulin resistance<br>-lipid : blood lipides and β hydroxy butyrate (HB)<br>-hormonal : cortiso, BDNF, IGF-1, IGFBP-1, IGFBP-3, HGH<br><br>Analyse of genes expression involved in :<br>-the circadian rhythm,<br>-longevity, life expectancy,<br>-autophagy,<br>-oxidative stress.	**Glycemic assessment**<br>-Morning : ↓ fasting glucose of 2 +/- 1 mg / dL (p = 0.02) and insulinemia of 2.9 +/- 0.4 mII / L (p <0.0001) and insulin resistance<br>-Evening : ↑ in insulin of 4.5 +/- 1.6 mIU / L (p = 0.01) and decrease in the evening HOMA-IR insulin resistance index by 1.09 +/- 0.43 (p = 0.02)<br>- ↓ of 24-hours glucose of 4 +/- 1 mg / dL (p = 0.0003) and reduction of hyperglycemic excursion by 12 +/- 3 mg / dL (p = 0.001).<br><br>**Lipid profile**<br>↑ LDL morning cholesterol of 9 +/- 4 mg / L (p = 0.02), HDL morning cholesterol of +/- 1 mg / dL (p = 0.03) and total cholesterol of 10 +/- 4 mg / dL (p = 0.04).<br>No increase in triglycerides (p = 0.29)<br>↑ of β hydroxy butyrate of 0.03 +/- 0.01 mM (p = 0.009)<br><br>**Hormonal balance**<br>Non-significant ↑ in morning cortisol levels of 1.5 +/- 0.9 µg / dL (p = 0.10) and decrease in evening cortisol levels of 1.4 +/- 0.6 µg / dL (p = 0.003),<br>↑ neurotrophic factor (BDNF) of 2.46 +/- 1.34 ng / mL (p = 0.09)<br>Non-significant decrease in IGF1 and IGFBP1 (p = 0.11)<br><br>**Gene expression**<br>-Circadian rhythm : modification of the expression of 6 genes out of 8<br>-Longevity : ↑ in morning expression of SIRT1 and MTOR in the evening<br>-Autophagy : ↑ in the expression of LC3A and ATG12<br>-Oxydative stress : No significative change in NOS3 expression (p=0.13)	**Strength**<br>Randomized crossover trial<br><br>Standardization of meals for the last 2 days<br><br>24-hours continuous glucose monitoring (CGM)<br><br>Objective measurement of energy expenditure<br><br>Biomolecular mechanisms exploration<br><br>**Limitation**<br>Small sample : lack of power<br><br>Short intervention that does not let the body the time to adapt its metabolism<br><br>Interpretation of data on gene expression is clinically limited.	HAS : 2.<br><br>Validity score : 23/25.

Time-Restricted Eating in women – A Pilot Study

Siobhan T. Smith, Jordan C. LeSarge, Peter W. R. Lemon

WURJ : Health and Natural Sciences, Volume 8, Issue 1 - Published the 18th Mars 2017.

	**Characteristics of the population**	**Study design**	**Main outcomes**	**Main results**	**Strength and limitations**	**Level of evidence**
**R3**	20 healthy adult women.<br><br>Mean age : 21.3 years ± 1.2 years.	4-week single-arm study.<br><br>Ontario, Canada.<br><br>**Protocol**<br>Delayed TRF 8/16 (from 12.00 p.m. to 8 p.m.)	Body composition by plethysmography before and after the intervention<br><br>Weekly self-assessment of adherence to the protocol as well as subjective feelings of hunger, satisfaction and fullness by VAS.	**Body composition**<br>↓ body mass of 0.6 +/- 1kg (p = 0.015)<br>↓ body fat in the 5 participants with regular physical activity (3 sessions per week or more) of 0.7 +/- 0.5 kg (p = 0.037)<br><br>**Adherence**<br>An average of 5.5 days of fasting per week with no difference across the weeks (p = 0.902)<br><br>**Subjective feeling and appetite**<br>No change in the feeling of hunger (p = 0.877) with average VAS of 44 mm<br><br>No change in satisfaction (p = 0.589) with an average VAS of 51 mm<br><br>Non change in fullness (p = 0.812) with an average VAS of 51 mm	**Limitation**<br>Non-controlled non-randomized study.<br><br>Healthy women : limits the generalization of results<br><br>Short intervention and small sample : lack of power.<br><br>No collection of eating habits before the intervention : confusion bias because the participants and inability to detect adaptation behaviors<br><br>No measurement of physical activity : risk of confusion bias<br><br>No measurement of food intake	HAS : 4.<br><br>Validity score : 17/25.

Early Time-Restricted Feeding Improves Insulin sensitivity, Blood Pressure, and Oxidative Stress even without Weight Loss in Men with Prediabetes<br>Elizabeth F. Sutton, Robbie Beyl, Kate S. Early, William T. Cefalu, Eric Ravussin, Courtney M. Peterson<br>*Cell Metabolism* 27, 1212–1221 - Published the 5th June 2018.						
	**Characteristics of the population**	**Study design**	**Main outcomes**	**Main results**	**Strength and limitations**	**Level of evidence**
**R4**	8 pre-diabetic overweight men.<br><br>Mean age : 59 ± 9 years.	Iso-caloric controlled trial, randomized of 5 weeks, in cross over with a wash out period of 7 weeks.<br><br>Pennington, Louisiana, United States of America.<br><br>**Protocol**<br>Early TRF (6/18), 3 meals from the participant's usual breakfast time.	Determination of biomarkers of glycemic and insulinemic metabolism, lipid balance and biological markers of inflammation and oxidative stress<br><br>Measuring cardiovascular risk factors<br><br>Self and hetero evaluation of the components of appetite by validated questionnaire and EVA.	**Glycemic metabolism**<br>↓ fasting insulin of 3.4 ± 1.6 mU / L (p = 0.05) and mean insulinemia of 26 ± 9 mU / L (p = 0.01) and insulin peak of 35 ± 13 mU / L (p = 0.01)<br><br>Improvement of insulin sensitivity of Langerhans beta cells by ↑ of the insulinogenic index of 14 ± 7 U / mg (p = 0.05) and<br><br>Improvement of insulin resistance by ↑ of the area under the curve of 3 36 ± 10 U / mg hours (p = 0.005).<br><br>**Oxidative stress marker**<br>↓ the level of 8-isoprostane of 11 +/- 5 pg / ml (p = 0.05).<br><br>**Cardiovascular risk factors**<br>↓ morning systolic blood pressure 11 +/- 4 mmHg (p = 0.03) and morning diastolic blood 10 +/- 4 mmHg (p = 0.03)<br><br>**Appetite assessment**<br>- ↓ the desire to eat 22 +/- 7 mm of EVA (p = 0.007) and the capacity to eat 23 +/- 6 mm of EVA (p = 0.001) in the evening, without modification of the feeling of hunger (p = 0.15).<br>- ↑ the feeling of fullness in the evening in the eTRF group by 31 +/- 6 mm (p <0.0001) and tends to increase the feeling of gastric fullness (p = 0.07).<br>- ↓ the morning value of the satiety hormone PYY of 23 +/- 7 pg / ml (p = 0.03) without significantly reducing the morning values of ghrelin (p = 0.41), the incretin GLP-1 (p = 0.26), adipokine leptin (p = 0.54) and high molecular weight Adiponectin (p = 0.61)<br><br>**Side effects**<br>Participants found more difficult to eat over 6 hours than to fast over 18 hours and found more feasible to eat on 7.8 +/- 1.8 h / 24h.<br>Non-significant ↑ in the morning heart rate from 5 +/- 3 bpm (p = 0.10) to 74 +/- 4 bpm<br>↑ morning triglycerides of 57 +/- 13 mg / dL (p = 0.0007) and total morning cholesterol (p = 0.02)	**Strength**<br>Randomized and controlled iso-caloric study with standardized meals<br><br>Measurement of biological markers of inflammation, oxidative stress, satiety and hunger<br><br>**Limitation**<br>Small sample : risk of lack of power<br><br>Pre-diabetic men : limits the generalization of results.<br><br>Longer fasting duration preceding testing (18 hr versus 12 hr in the control arm) could underestimate the insulin control and disturb the lipid balance.<br><br>Punctual measurement of glucose<br><br>No measure of physical activity	HAS : 2.<br><br>Validity score : 23/25.

Early Time-Restricted Feeding Reduces Appetite and increases Fat oxidation But Does Not Affect Energy expenditure in Humans

Eric Ravussin, Robbie A. Beyl, Eleonora Poggiogalle, Daniel S. Hsia, and Courtney M. Peterson

Obesity (Silver Spring). 1244-1254 – Published the 28th August 2019.

	Characteristics of the population	Study design	Main outcomes	Main results	Strength and limitations	Level of evidence
**R5**	11 healthy adults.<br><br>Mean age : 32 years.	4-day randomized crossover controlled trial with a wash out period of 3.5 to 5 weeks.<br><br>Pennington, Louisiana, United States of America.<br><br>**Protocol**<br>Early TRF (6/18) with meals at 8.00 a.m, 12.00 p.m and 2.00 p.m.<br>-days 1-2 : free meals.<br>-days 3-4 : standardized and partially supervised meals at the research center.	Measurement of energy expenditure on day 4 in a calorimetric chamber.<br><br>Subjective measurement of appetite sensation, energy level, arousal state and body temperature by validated questionnaire and VAS.<br><br>Dosage of blood and urine markers of the hormones of satiety and hunger.	**Energy consumption and expenditure**<br>No increase in 24-hour energy expenditure.<br>↑ fat and lean body oxidation.<br><br>**Appetite**<br>↓ several aspects of appetite in the middle of the day (11 am-5 p.m; p <0.05)<br>-improving hunger indicators in the evening (10:30 p.m .; p <0.007)<br>- tends to ↓ the average desire to eat by 5 +/- 2 (p = 0.08), the amplitude of hunger during the day by 10 +/- 3 (p = 0.006) and the desire to eat by 9 + / - 5 mm (p = 0.09)<br>- tends to ↑ the feeling of general fullness by 3 +/- 2 mm (p = 0.10) and gastric by 3 +/- 2 mm (p = 0.06).<br><br>**Biological appetite markers**<br>- Morning : ↓ ghrelin of 43 +/- 15 pg / mL (p = 0.009), leptin of 4 +/- 1 ng / mL (p = 0.01) and GLP-1 of 0.8 +/- 0.3 pmol / mL (p = 0.008)<br>- Evening : ↑ in PYY (satiety) of 17 +/- 6 pg / mL (p = 0.02) and tendency to decrease ghrelin by 22 +/- 12 pg / mL (p = 0.09)<br>- ↓ average ghrelin of 32 +/- 10 pg / mL (p = 0.006) and tends to increase average leptin (p = 0.07).	**Strength :**<br>Randomized controlled iso caloric study<br><br>Standardization and partial supervision of the energy intake<br><br>Objective measurement of energy expenditure in a calorimetric chamber<br><br>**Limitation**<br>No body composition measurement to assess whether eTRF impacts lean and fatty mass.<br><br>Small sample size and short intervention : lack of power.<br><br>Test designed to detect a difference in energy expenditure of 80kcal / d (20 to 40 in the study) : lack of power<br><br>No collection of baseline habits : no detection of any adaptation behaviors	HAS : 2.<br><br>Validity score : 22/25.

Effect of 8-hour time-restricted feeding on sleep quality and duration in adults with obesity<br>Kelsey Gabel, Kristin K. Hoddy, Helen J. Burgess, Krista A. Varady<br>*Applied Physiology, NuritION, and Metabolism* - 2019, 44(8): 903-906 – Published the 20th February 2019.						
	**Characteristics of the population**	**Study design**	**Main outcomes**	**Main results**	**Strength and limitations**	**Level of evidence**
**R6**	23 obese adults.<br><br>Mean age : 50 ± 2 years.	12-week single-arm trial.<br><br>Chicago, United States of America.<br><br>**Protocol**<br>Delayed TRF 8/16 with ad libitum meal from 10.00 a.m h to 6.00 p.m<br><br>Free meals<br><br>2-week baseline period to collect diet and physical activity habits	Anthropometric measurement and measurement of body composition by Dual-energy X-ray Absorptiometry (DXA)<br><br>Measurement of program adherence by a 7-day food recording in diet log in weeks 1 and 12<br><br>Physical activity measurement by podometry during the week 1 and 12<br><br>Sleep quality measurement by several questionnaires (insomnia, sleep quality, free questionnaire, OSA screening)<br><br>Constitution of 3 groups according to the quality of their sleep:<br>-all sleepers (n = 23)<br>-good sleepers (n = 13)<br>- bad sleepers (n = 10)	**Adhrence**<br>80% adherence in "all sleepers", 83% in "good sleepers" and 76% in "bad sleepers"<br><br>Physical activity : unchanged in the 3 groups<br><br>**Anthropometric measurement and body composition**<br>↓ 4% weight ($p < 0.001$) and fat mass (5%) ($p < 0.01$) with no difference in the level of physical activity<br><br>**Sleep quality**<br>No improvement or deterioration in the quality of sleep.	**Strength :**<br>Objective measurement of physical activity by podometer for 2 weeks (the first and the last)<br><br>**Limitation**<br>Controlled non-randomized study<br><br>Sample size designed to detect weight loss but not powerful enough to detect a change in sleep quality<br><br>Inconsideration of sleeping habits and therefore of any secondary compensation<br><br>Subjective measurement of food intake without caloric counting : risk of classification bias<br><br>Measurement of physical activity only on two weeks	HAS : 4.<br><br>Validity score : 20/25.

Effects of 8-hour time restricted feeding on body weight and metabolic disease risk factors in obese adults : A pilot study<br>Kelsey Gabel, Kristin K. Hoddy, Nicole Haggerty, Jeehee Song, Cynthia M. Kroeger, John F. Trepanowski, Satchidananda Panda, and Krista A. Varady,<br>*Nutrition and Healthy Aging* 4 (2018) 345–353 - Published the 15th June 2018.						
	**Characteristics of the population**	**Study design**	**Main outcomes**	**Main results**	**Strength and limitations**	**Level of evidence**
**R7**	23 healthy obese adults.<br><br>Mean age : 50 ± 2 years.	12-week, non-randomized, matched historical group controlled trial.<br><br>Chicago, United States of America.<br><br>**Protocol**<br>Delayed TRF 8/16 with ad libitum meal from 10.00 a.m h to 6.00 p.m<br><br>Free meals<br><br>2-week baseline period to collect diet and physical activity habits<br><br>Control to a group from a historical cohort by matching on body weight and metabolic disease risk factors	Measurement of program adherence by a 7-day food recording in diet log in weeks 1 and 12<br><br>Measurement of physical activity by podometry in weeks 1 and 12<br><br>Weekly weighing and body composition measurement by DXA<br><br>Measurement of metabolic disease risk factors at the baseline period and week 12 : blood pressure, heart rate, blood sample (blood lipids, glucose and insulin).	**Adherence to protocol**<br>Average compliance of 5.6 days / week with reduction of the food window to 8 +/- 1 hours<br><br>Dropout rate high of 26% (n = 6) but not linked to the dietary protocol<br><br>↓ caloric intake of 350kcal / d (p=0.04) without change in the distribution between protein, carbohydrates and fat<br><br>No change in physical activity<br><br>**Body composition**<br>↓ 2.6% relative weight,<br>↓ of relative BMI with no difference in body composition<br><br>**Risk factor for metabolic disease**<br>↓ systolic blood pressure of 7 +/- 2 mmHg (p = 0.02)	**Strength**<br>Objective measurement of physical activity by pedometer for 2 weeks (the first and the last)<br><br>**Limitation**<br>Non-randomized controlled trial to a historical group whose recruitment period could potentially differ from 5 years.<br><br>Measurement of physical activity for only 2 weeks : risk of confusion bias<br><br>Subjective measurement of food intake : risk of classification bias<br><br>Obese population : limits the generalizabiliy of the results	HAS : 4.<br><br>Validity score : 18/25.

Effects of eight weeks of time-restricted feeding (16/8) on basal metabolism, maximal strength, body composition, inflammation, and cardiovascular risk factors in resistance-trained males

Tatiana Moro, Grant Tinsley, Antonino Bianco, Giuseppe Marcolin, Quirico Francesco Pacelli, Giuseppe Battaglia, Antonio Palma, Paulo Gentil, Marco Neri and Antonio Paoli

Journal of Translational Medicine volume 14, Article number : 290 (2016) - Published the 13th October 2016.

	Characteristics of the population	Study design	Main outcomes	Main results	Strength and limitations	Level of evidence
**R8**	34 adult men who strength train.<br><br>Mean age : 29.21 ± 3.8 years.	8 week randomized controlled trial.<br><br>Venice, Italy.<br><br>**Protocol**<br>TRF+RT (resistance training) vs RT alone<br><br>TRF : dTRF 8/16 with 3 meals at 12.00 p.m, 4.00 p.m and 8.00 p.m everyday<br><br>RT : 3 non-consecutive day of training sessions per week supervised by the research team	Anthropometric and body composition measurement by DXA<br><br>Muscle measurements :<br>-muscle volume by calculating the cross-sectional area<br>-legs and arms circumference<br>-muscle strength by 1-RM calculating (one repetition maximum)<br><br>Metabolic health biomarkers :<br>-glucose, insulin and calculating of the HOMA-IR (insulin resistance index)<br>-blood lipids<br>-blood hormones : testosterone, IGF1, TSH, T3<br>-adiponectin and leptin levels<br>-inflammation factors : inter leukine-6 (IL), IL-1 β and TNF α,<br><br>Measurement of energy expenditure during training sessions by calometry (respiratory ratio, resting energy expenditure)<br><br>Measurement of food intake ; validated 7-day food diary and a weekly structured interview by a dietician	No difference in caloric intakes between groups<br><br>**Body composition**<br>↓ body fat in TRF group of 16.4% against 2.8% in the control group (p = 0.0448)<br>-maintainance of lean body mass in the two groups (+0.86 vs + 0.64%).<br><br>**Muscle measurements**<br>-conservation of limbs muscles volume in the two groups<br>-↑ muscle strength in legs with no difference between the two groups<br><br>**Biological markers**<br>-↓ blood glucose levels (p = 0.0001) and insulin (p = 0.0303) in the TRF group<br>-↓ of HOMA-IR index in TRF group : improvement of insulin resistance<br><br>-no change in the blood lipids except ↓ triglycerides in TRF group (p = 0.00052)<br><br>- ↓ IGF1 and testosterone in TRF group<br><br>- ↑ adiponectin and ↓ leptin (results which were no longer significant after normalization on fat mass)<br><br>- ↓ isolated from T3<br><br>- ↓ TNF α (p = 0.0001) and IL-1β (p = 0.0042) in TRF group<br><br>**Energy expenditure**<br>↓ respiratory ratio in TRF group (p = 0.0421), which indicates lipid oxidation	**Strength :**<br>Randomized controlled study<br><br>Energy expenditure measurement by calometry during training sessions<br><br>**Limitation**<br>Subjective measurement of caloric intake : risk of classification and confusion bias<br><br>No measurement of energy expenditure outside training sessions<br><br>Young healthy men population : limits the generalizabiliy of the results	HAS : 2.<br><br>Validity score : 23/25.

Safety of 8-h time restricted feeding in adults with obesity.<br>Kelsey Gabel, Kristin K. Hoddy, Krista A. Varady<br>*Applied Physiology, Nurition, and Metabolism* - 2019 Jan ;44(1) :107-109 - Published the 14th September 2014.						
	**Characteristics of the population**	**Study design**	**Main outcomes**	**Main results**	**Strength and limitations**	**Level of evidence**
**R9**	23 Healthy obese adult.<br><br>Mean age : 50 ± 2 years.	12-week singe-arm trial.<br><br>Chicago, United States of America.<br><br>**Protocol :**<br>Delayed TRF 8/16 with ad libitum meal from 10.00 a.m h to 6.00 p.m<br><br>Free meals<br><br>2-week baseline period to collect diet and physical activity habits	Semi quantitative measurement of energy intake by 7 days food recording during the baseline period and week 12.<br><br>Measurement of physical activity using a podometer 7 days to week 1 and 12.<br><br>Measurement of resting energy expenditure by indirect calometry.<br><br>Measurement of protocol side effects by questionnaires relating to gastrointestinal and neuropsychological effects, eating disorder symptoms (MEADS questionnaire validated), body shape questionnaire (BSQ), dietary restraint and emotional eating (TFEQ)<br><br>Biological analysis at weeks 1 and 12: CBC; β-hydroxy butyrate	**Body composition**<br>↓ relative weight of 2.6 +/- 0.5% (p <0.001)<br><br>**Energy exchanges**<br>↓ caloric intake (p=0.04) without change in the distribution between protein, carbohydrates and fat<br><br>No change in physical activity and resting energy expenditure<br><br>**Side effects**<br>No significant change in questionnaires responses<br><br>**Biological measurements**<br>No change in the biological markers analyzed**.**	**Limitation**<br>Non-controlled study<br><br>Small sample size : lack of power<br><br>Intervention duration too short for measuring metabolic changes<br><br>Subjective measurement of energy intake<br><br>No intermediate measurement in the half of the protocol<br><br>Measurement of energy expenditure by MedGEM calorimeter known to overestimate rest energy expenditure	HAS : 4.<br><br>Validity score : 18/25.

Time Restricted Feeding on Overweight, Older Adults : A Pilot Study<br>Stephen D. Anton, Stephanie A. Lee, William T. Donahoo, Christian McLaren, Todd Manini, Christiaan Leeuwenburgh and Marco Pahor<br>*Nutrients* 2019, 11, 1500 - Published the 30th June 2019						
	**Characteristics of the population**	**Study design**	**Main outcomes**	**Main results**	**Strength and limitations**	**Level of evidence**
**R10**	10 overweight elderly adult.<br><br>Mean age : 77.1 years.	4-week single-arm trial.<br><br>Gainesville, Florida, United States of America.<br><br>**Protocol**<br>TRF 8/16	Adherence measurement by weekly telephone interview<br><br>Metabolic markers measures : weight, waist circumference, fasting glucose, blood pressure<br><br>Measure of cognitive function by MoCA score<br><br>Physical function measurement : speed test on 6 min walk as well as grip strength by a dynamometer<br><br>Tolerance measurement : quality of life questionnaire (HRQoL), on mental and physical fatigability by validated Pittsburgh questionnaire	**Adherence**<br>84% mean adhesion with an average fasting period of 15.8 hours/day.<br><br>**Body composition**<br>Mean weight loss of 2.6 kg ($p < 0.01$)<br><br>**Physical and mental function**<br>Non-significant improvement in physical and mental functions and quality of life<br><br>**Side effects**<br>Minor side effects with headache ($n = 2$) and dizziness ($n = 1$) resolving after food and water, respectively.	**Limitation**<br>Controlled non-randomized study<br><br>Measurement of weight without body composition assessment : major risk of measurement bias with inability to distinguish the type of lost mass (fat, lean, water)<br><br>Small sample size : lack of power<br><br>Subjective measurement of energy intake timing by self declaration with no colorie counting: risk of classification bias<br><br>Overweight elderly population : limits the generalizabiliy of the results<br><br>**Strength**<br>Adhesion threshold defined a priori : if participant fasts at least 14 hours a day for 2 weeks<br><br>Measurement of relevant morbidity and mortality markers in elderly population	HAS : 4.<br><br>Validity score : 17/25.

Time-Restricted Feeding Improves Glucose Tolerance in Men at Risk for Type 2 Diabetes : A Randomized Crossover Trial Amy T. Hutchison, Prashant Regmi, Emily N.C. Manoogian, Jason G. Fleischer, Gary A. Wittert Satchidananda Panda, and Leonie K. Heilbronn *Obesity* (2019) 27, 724-732 - Published the 19th April 2019						
	**Characteristics of the population**	**Study design**	**Main outcomes**	**Main results**	**Strength and limitations**	**Level of evidence**
**R11**	15 pre-diabetic men <br>Risk of diabetes estimated by the AUSDRIK score. <br>Mean age : 55 ± 3 years.	One week crossover randomized trial with a 2 week wash out period. <br>Adelaide, Australia. <br>**Protocol** eTRF 9/15 (8.00 a.m to 5.00 p.m) vs. dTRF (12.00 p.m to 9.00 p.m) <br>Control to the baseline period and TRF groups between each others <br>Meal test : ingestion of a mixed-nutrient liquid test	Measurement of body composition by DXA, measure of body weight, waist and hip circumference <br>Measure of blood pressure <br>Biological measurements of fasting glucose, continuous glucose monitorage (CGM) and blood lipids over 7 days <br>Measuring of : -markers of appetite by VAS -gastric emptying time <br>Biological markers of gastrointestinal hormones involved in appetite (ghrelin, peptide YY, GLP1, GIP, amylin) <br>Measurement of energy expenditure by a sensory monitor worn in an armband <br>Self declaration of food timing in a food log	**Body composition** Weight loss with no difference between groups (p> 0.66) <br>**Glycemic and lipid metabolism** ↓ of glucose AUC of 36% in eTRF against 21% for dTRF (p = 0.002) and tendency to decrease the insulin AUC in the TRF groups (p = 0.09) <br>↓ mean fasting glucose in the eTRF group compared to the baseline period (p=0.02) but not compared to dTRF (p=0.17) <br>↓ fasting triglycerides (p = 0.003) in the two TRF groups <br>**Appetite** No effects of TRF on appetite hormones or on the hunger feeling, fullness and the desire to eat. ↓ GLP1 (p = 0.02) for TRF groups ↑ overall feeling of fullness for eTRF (p = 0.038) <br>**Energy expenditure** No difference in energy expenditure	**Limitation** Prediabetic population : limits the generalizabiliy of the results <br>Short duration of the study and small sample size : lack of power <br>No measurement of food intake <br>Subjective measurment of food timing <br>**Strength** Objective measurement of physical activity <br>Continuous glucose monitorage for 7 days	HAS : 2. <br>Validity score : 23/25.

Time-restricted feeding in young men performing resistance training : A randomized controlled trial<br>Grant M. Tinsley, Jeffrey S. Forsse, Natalie K. Butler, Antonio Paoli, Annie A. Bane, Paul M. La Bounty, Grant B. Morgan & Peter W. Grandjean<br>*European Journal of Sport Science*, 2016 – Published the 24th August 2016						
	**Characteristics of the population**	**Study design**	**Main outcomes**	**Main results**	**Strength and limitations**	**Level of evidence**
**R12**	18 young adult men who strength train.<br><br>Mean age : 22 ± 2.4 years.	8 week randomized controlled trial.<br><br>Texas, United States of America.<br><br>**Protocol**<br>TRF+ RT vs RT alone<br><br>TRF 4/20 from 4 p.m. to 8.00 p.m. without calorie restriction 4 d/w. on the 4 non-workout days<br><br>RT: 3 non-consecutive day of training sessions per week at the gym with monitoring of adherence by a food diary	Program tolerance measures by VAS<br><br>Measurement of adherence to exercise program by training log<br><br>Measure of adherence to diet protocol by food log.<br><br>Measurement of body composition by DXA.<br><br>Measurement of muscle volume by ultrasound<br><br>Muscle strength testing by calculating 1-RM (one repetition maximum) and endurance by repetition until 65% failure of 1-RM.	**Adherence to TRF**<br>95% compliance with the TRF program : some participants report greater difficulty on weekends and after several days.<br><br>Difficulty at 3 mm stable throughout the 8 weeks.<br><br>↓ energy consumption of 650 kcal / d on average between fasting days and non-fasting days<br><br>↓ significant weekly energy consumption between the TRF-RT group and the control group (p = 0.01) with a higher consumption of proteins (p = 0.03), fewer carbohydrates (p = 0.005) and in trend lower in fat (p = 0.30).<br><br>**Body composition**<br>No significant change in weight (p = 0.38) and fat mass (0.32)<br><br>**Muscle performance**<br>-no significant change in lean body mass (p = 0.49)<br>-↑ in the muscle volume of the thighs and arms in the 2 groups without differences<br>-↑ in maximum strength and muscular endurance in the 2 groups without difference.	**Strength**<br>Randomized controlled trial<br><br>Intermediate measure at week 4<br><br>**Limitation**<br>No direct supervision of the training protocol and no measurement of physical activity outside of exercise sessions : confusion bias<br><br>Small sample size : lack of power<br><br>No standardization and subjective measurement of energy intake : risk of confusion bias	HAS : 2.<br><br>Validity score : 19/25.

Time-restricted feeding influences immune responses without compromising muscle performance in older men

Maha Gasmi Ph.D., Maha Sellami Ph.D., Joshua Denham Ph.D. d, Johnny Padulo Ph.D., Goran Kuvacic PhD, Walid Selmi Ph.D., Riadh Khalifa Ph.D.M. Gasmi et al.

Nutrition 51-52 (2018) 29–37 - Published the 4th December 2017.

	Characteristics of the population	Study design	Main outcomes	Main results	Strength and limitations	Level of evidence
**R13**	20 years old men (n=20) and 50 years old men (n=20)	12 week randomized controlled trial.<br><br>Tunisia.<br><br>**Protocol**<br>4 groups :<br>TRF 12/12 50 years<br>TRF 12/12 20 years<br>Control group 50 years<br>Control group 20 years<br><br>Protocol of 2 d/ w. separated by 48 hours<br><br>Nightly TRF with fast from sunrise to sunset	Measurement of adherence to the food protocol by weekly phone interview and recording food intake by participants in diet diary<br><br>Anthropometric measurement and muscular function testing by six 35m sprints with assessment of speed, time and developed force,<br><br>Measurement of blood pressure<br><br>Biological assay : CBC, immunity cells	**Adherence to TRF**<br>No difference in energy intake<br><br>**Body composition**<br>No change in body mass, fat and lean mass between groups ($p > 0.05$)<br><br>**Muscle performance**<br>No change in markers of muscle function in young and old groups compared to control groups<br><br>**Biological markers**<br>Red blood cells ($p < 0.001$) and hemoglobin ($p = 0.043$) were lower in the 50-year-old group at the beginning of the protocol and these differences were not noted after the TRF protocol ($p > 0.05$)<br><br>White blood cells were higher in young participants at the start of the protocol ($p = 0.026$) and this difference persisted at the end of the study ($p = 0.035$)<br><br>↓ of white blood cells in the TRF groups ($p < 0.05$)<br><br>Neutrophils were higher at the start of the study in the youth groups ($p = 0.03$) and this difference disappeared in the TRF groups ($p > 0.05$)<br><br>↓ significant in neutrophils cells in the TRF groups ($p < 0.05$)<br><br>Lymphocytes were higher in young people ($p < 0.001$) and this difference disappeared in the TRF groups ($p > 0.005$) without change in T3 and T4 lymphocytes<br><br>↓ NKCD56 lymphocytes in the elderly ($p = 0.048$) and young group ($p < 0.001$) and NKCD16 only in the young ($p < 0.001$)	**Strength :**<br>Randomized controlled trial<br><br>Pairing according to caloric intake<br><br>Study duration (3 months) and sample size<br><br>**Limitation**<br>Subjective measurement of energy intake<br><br>Male population : limits the generalizabiliy of the results<br><br>No physical activity measurement : risk of confusion bias	HAS : 2.<br><br>Validity score : 23/25.

Time-restricted feeding plus resistance training in active females : A randomized trial<br>Grant M Tinsley, M Lane Moore, Austin J Graybeal, Antonio Paoli, Youngdeok Kim, Joaquin U Gonzales, John R Harry, Trisha A VanDusseldorp, Devin N Kennedy, and Megan R Cruz<br>*Am J Clin Nutr* 2019 ;110 :628–640 - Published the 3th July 2019.						
	**Characteristics of the population**	**Study design**	**Main outcomes**	**Main results**	**Strength and limitations**	**Level of evidence**
**R14**	40 healthy women who strength train.<br><br>Target age : 18-30 years.<br><br>.	8 week randomized controlled trial.<br><br>Lubbock, Texas, United States of America.<br><br>**Protocol**<br>Randomization in 3 groups :<br>-RT + placebo<br>-TRF + RT + placebo<br>-TRF + RT + HMB<br><br>Delayed TRF 8/16 (12.00 p.m to 8.00 p.m) everyday<br><br>HMB : β-hydroxy methyl butyrate supplementation<br><br>RT : 3 non-consecutive day of training sessions per week supervised in the research laboratory<br><br>Measurement of energy expenditure outside training sessions.	Body composition measurement by DXA<br><br>Measurement of physical activity outside training sessions by accelerometer<br><br>Measurement of resting energy expenditure and the substrates oxydation by indirect calometry<br><br>Vascular function assessment : blood pressure, heart rate, index of arteriel stiffness<br><br>Subjective measurement of adherence, sleep, menstrual cycle and diet by standardized questionnaires<br><br>Muscle volume measurement by ultrasound<br><br>Muscle strength testing by calculating 1-RM (one repetition maxium) and muscular endurance by repetitions until failure with 65% of 1-RM<br><br>Biological assays<br>-blood glucose, lipids and cortisol<br>-salivary and urinary cortisol.	**Adherence to TRF**<br>↓ caloric intake window to 7.5 hours a day<br><br>**Energy balance**<br>↑ energy intakes from 20 to 200 kcal / d in the 2 TRF groups<br>- no difference in energy expenditure, substrates oxydation, biological and vascular markers<br><br>**Body composition**<br>↑ lean mass, muscle volume of arms and legs in the 3 groups<br>↓ body fat for the 2 TRF groups in the per protocol analysis of 4%-7%<br><br>**Muscle performance**<br>Improvement in muscle performance (strength and endurance) in all groups<br><br>**Side effects**<br>-no effects in 84% of the participants at half of the protocol, and in 90% at the end of the protocol<br>-no disturbance of the menstrual cycle.	**Strength**<br>Randomized, double-blind, controlled trial for supplementation<br><br>Sample size and duration of the study<br><br>Direct supervision of physical activity during exercise<br><br>Measurement of energy expenditure outside training sessions<br><br>**Limitation**<br>Subjective measurement of energy intake<br><br>Female population : limits the generalizabiliy of the results	HAS : 2.<br><br>Validity score : 23/25.

A Smartphone App Reveals Erratic Diurnal Eating Patterns in Humans that Can Be Modulated for Health Benefits<br>Shubhroz Gill and Satchidananda Panda.<br>*Cell Metabolism* 22, 789–798. Published the 3th November 2015.						
	**Characteristics of the population**	**Study design**	**Main outcomes**	**Main results**	**Strength and limitations**	**Level of evidence**
**R15**	8 healthy obese adults.<br><br>Target age : 18 years or more.	16-week single-arm trial.<br><br>La Jolla, California, United States of America.<br><br>**Protocol**<br>Measurement of eating habits by a smartphone app during 3 weeks<br><br>TRF 10/14 protocol every day for 16 weeks	**Measurement (carried out at the baseline period, 16 weeks then at 1 year)**<br><br>Anthropometric measurements<br><br>Smarphone app-based caloric intake monitoring (mCC) during 3 weeks before the intervention and then during the 16 weeks protocol<br><br>Subjectives mesures :<br>Self-assessment of energy level, hunger at bedtime and sleep quality by VAS	**Preleminary period**<br>-mean feeding period : 15h<br>-mean BMI of 31.77 kg / m² for men, 34.91 kg / m² for women.<br>-40% of calories are consumed after 6 p.m.<br>-100% of the daily maintenance caloric intake was taken at 6.30 p.m.<br>-metabolic jetlag concept : variation in breakfast time between working/week days and free/weekend days of 1 hour and more in 65% of participants.<br><br>**TRF effects**<br>- average ↓ of caloric intake window by 4hr35 ($p < 0.001$) for all participants<br>- ↓ metabolic jetlag <1 hour<br>- ↓ daily calorie intake of 20.26% ($p < 0.05$)<br>- ↓ of relative weight by 4% ($p < 0.05$)<br>- ↓ BMI by 1.15 kg / m² ($p < 0.05$)<br><br>-significant improvement in sleep quality (VAS went from 5.5 mm to 7 after 16 weeks, then 8 mm 1 year after the start of intervention)<br>-↑ morning energy (VAS went from 5.5 to 7 then 8 after 1 year) and overall energy<br>-↓ of hunger (6.3 mm to 5.1 mm after 16 weeks)	**Strength**<br>Smartphone app allowes a sensitive measurement of caloric intake<br><br>Positive results (weight loss, improved sleep, feeling of energy) maintained at 1 year after the study<br><br>**Limitation**<br>Smartphone application : poor adhesion for small snacks, the transfer of portion size is subjective with false negative rate of 10%<br><br>Small sample size : lack of power<br><br>No body composition measurement : risk of measurement bias and inability to distinguish the type of lost mass (fat, lean, water)<br><br>No measurement of energy expenditure : risk of confusion bias	HAS : 2.<br><br>Validity score : 21/25.

Determinants of Adherence in Time-Restricted Feeding in Older Adults : Lessons from a Pilot Study

Stephanie A. Lee, Caroline Sypniewski, Benjamin A. Bensadon, Christian McLaren, William T. Donahoo, Kimberly T. Sibille and Stephen Anton

Nutrients 2020, 12, 874. Published : 24 March 2020 ;

	Characteristics of the population	Study design	Main outcomes	Main results	Strength and limitations	Level of evidence
**R16**	10 overweight, sedentary, older adults (6 women and 4 men).<br><br>Mean age : 77.1 yeas.	4-week single-arm pilot study.<br><br>Gainesville, Florida, United States of America.<br><br>**Protocol**<br>TRF 8/16 every day with self-selection of fasting and eating window that can varies each day<br>-day 1-3 : 12-14 hr fast<br>-day 4-6 : 14-16 hr fast<br>-day 7-28 : 16h fast	**Feasability - Safety**<br>Weekly phone interviews<br><br>Exit interview<br><br>Diet satisfaction survey including questions on biological, psychological and socio-environmental factors<br><br>**Adherence**<br>Self-reported by daily eatin time log	**Feasability**<br>90% of the participants (n=9) completed the protocol<br><br>Few adverse events : headaches (n=2) and dizziness (n=1) resolved after hydratation and feeding<br><br>**Adherence**<br>Mean self-reported adherence was 84%<br>Average time eating period was 10 :20 a.m to 6 :40 p.m<br>6 participants didn't understand the regimen<br>-3 reported consuming snacks during fasting periods<br>-2 confused low with no-calorie food<br>-1 ate only one meal a day<br><br>**Diet satisfaction servey responses (n=9) :**<br>-protocol was not difficult in 7 participants and 6 declared that fasting got easier over the study period<br>-6 participants declared they can maintain TRF during 6 months, and 5 stated they would continue fasting after the study<br>-only one participant reported hunger, but he was the one that ate only one meal in the day<br>-sleep patterns unaffected in 8 participants<br>-energy levels retained stable in 8 participants<br>-mood and quality of life unaffected in 6 patients	**Strength**<br>Adherence daily recorded in an eating time log<br>Weekly phone calls to check adherence and adjustes the protocol<br><br>**Limitation**<br>Single-armed pilot study<br><br>Only 4 participants completed all three weekly phone calls<br><br>Self-reported adherence with 6 participants that didn't fully understand the regimen : risk of measure bias<br><br>No measure of food intake<br><br>Small sample size and short intervention duration : lack of power<br><br>Elder overweigth population : limits the generalizability of the results	HAS : 4<br><br>Validity score : 19/20

Adherence to Time-Restricted Feeding and Impact on Abdominal Obesity in Primary Care Patients : Results of a Pilot Study in a Pre–Post Design

Dorothea Kesztyüs, Petra Cermak, Markus Gulich and Tibor Kesztyüs

Nutrients 2019, 11, 2854. Published : 21 November 2019

	Characteristics of the population	Study design	Main outcomes	Main results	Strength and limitations	Level of evidence
**R17**	40 abdominally obese adults.<br><br>Mean age : 49. 1 ± 12.4 years.	12-week single-arm pilot study.<br><br>Helmholzstr, Germany.<br><br>**Protocol**<br>TRF 8/16 every day for 12 weeks with self-selection of the food intake period by the participant<br><br>In general practioner's ocffice	**Adherence**<br>Proportion of days fasted/ total number of days recorded in a diet diary<br><br>Phone intervieuw 2-3 weeks after the beginning of the protocol<br><br>Questionnaires on : feasibilty, effects and side effects, physical activity and screen-media consumption<br><br>**MetS markers at the baseline and at 3 months**<br>Body weight, height and determination of the BMI<br>Waist circumference (WC) and calculation of waist-to height ratio (WHtR) as proxies for intra-abdominal fat ((WHtR ≥ 0.5 : abdominal obesity).<br><br>Blood parameters<br>LDL, HDL, triglycerides, HbA1c, hsCRP	**Adherence**<br>86 ± 15% adherence : fasting target acieved on all days recorded<br>Mean time eating period was 10.30 a.m to 18.05 p.m<br>Reduction of sweet and salty snacks (p<0.001)<br><br>**Side effets**<br>58% felt hungry once a week or less and only 8% felt hungry every day<br>13% participants experienced side effects such as cravings, dizziness and nausea at the beginning and 8 reported improvements over time<br>63% noticed any side effects<br>40% found the protocol easy or very easy<br>76% felt positive of very positive<br><br>**MetS markers**<br>↓ weight of 1.7 ± 2.5 kg (p<0.001)<br>↓ BMI of 0.6 ± 0.9 kg/m² (p<0.001) with 5 participants who fall under the BMI threshold of 25<br>↓ WC -5.3 +/- 3.2 cm (p<0.001)<br>↓ WHtR of 0.03 +/- 0.2 (p<0.001) with 3 participants no longer classified as abdominally obese<br><br>↓ HbAc1 by 1.4 ± 3.5 mmol/mol (-3.8%) (p=0.003)	**Strength**<br>Low rate of dropout (5%)<br><br>Good adherence of the participants<br><br>**Limitation**<br>Non comparative non randomized pilot study<br><br>Men were underrepresented : limits the generalizability of the results<br><br>Self-reported hours of eating<br><br>No measure of food intake	HAS : 4<br><br>Validity score : 21/25

Ten-Hour Time-Restricted Eating Reduces Weight, Blood Pressure, and Atherogenic Lipids in Patients with Metabolic Syndrome<br>Michael J. Wilkinson, Emily N.C. Manoogian, Adena Zadourian, Saket Navlakha, Satchidananda Panda, Pam R. Taub<br>*Cell Metabolism* 31, 1–13 - Published the 7th January, 2020						
	**Characteristics of the population**	**Study design**	**Main outcomes**	**Main results**	**Strength and limitations**	**Level of evidence**
**R18**	19 participants with MetS<br><br>Mean age : 59 +/- 11 years	12-week single-arm study<br><br>La Jolla, California, United States of America.<br><br>**Protocol**<br>TRF 10/14 every day with self-selection<br><br>« add-ons » statin and anti-hypertensive medications	**Adherence and food intake** by a smartphone app (mCC)<br><br>**Sleep** duration by smartphone app and sleep quality bu PSQI<br><br>**Physical activity** by actigraphy devices (actiwatch) during the 1st andi last week<br><br>Weight, BMI, body composition, WC, Viscral fat rating, BP<br><br>**Biomarkers**<br>-24hr CGM during the 1st and last week, HbA1c, fasting insulin, HOMA-IR<br>-Lipids<br>-hs-CRP, ALT, AST, TSH, CBC	**Adherence**<br>Baseline eating window was 15.13 ± 1.13h reduced to 10.78 ± 1.18h (-4.35 ± 1.32 h)<br>Mean adherence of 85 ± 12%<br>63.2% were somehow engaged in TRF at 16 ± 4 months<br>No major side adverse events<br><br>**Sleep**<br>↑ in sleep duration by 12.45 min (p=0.302)<br>↑ in sleep durant and efficiendy in 84% of participants<br>↓ in variance in wake time by 35% (p=0.035)<br>↓ in variance of the time of first caloric intake of 40% (p=0.001) and the time of last caloric intake by 44% (p=0.0001)<br>↑ morning restfulness by 23% (p=0.019)<br>Trend in ↑ sleep quality based on PSQI (p=0.0164)<br><br>**Caloric intake**<br>↓ by 8,62% ± 14.47% (p=0.007)<br><br>**Physical activity** inchanged with a trend in reduction in physical activity (p=0.069)<br><br>**Risk factors for CVD**<br>↓ body weight (-3%) (p=0.00028)<br>↓ BMI (-3%) (p=0.00011)<br>↓ body fat (-3%) (p=0.00013)<br>↓ visceral fat rating (-3%) (p=0.004)<br>↓ WC (- 4.46 ± 6.72 cm, p=0.0097) correlated with decreased eating interval (p=0.005)<br><br>↓ total cholesterol (-7%) (p=0.03) ↓ LDLc (-11%) (p=0.016)<br>↓ non-HDLc (-9%) (p=0.04) - Not attribuable to weight changes<br>↓ systolic and diastolic BP (p=0.041, p=0.004, respectively)<br>Synergic effects in participants who take statin and anti-hypertensive therapy<br>Trend ↓ in fasting glucose (p=0.081), HbA1c (0.058), CGM (p=0.19)	**Strength**<br>Caloric intake measure in free-living conditions by smartphone app<br><br>Physical activity measure by actigraphy<br><br>CGM<br><br>**Limitation**<br>Single-arm, unblindedn, unrandomised pilot study<br><br>Smal sample size : lack of power<br><br>Participant with MetS : limits the generalizability of results<br><br>The smartphone app for measuring food intake stills less precise than iso-caloric, standardised-food intake study, with 10%	HAS : 2<br><br>Validity score : 21/25

Time-restricted feeding improves markers of Cardiometabolic health in physically active college-age men : A 4-week randomized pre-post pilot study<br>Matthew J. McAllister, Brandon L. Pigg, Liliana I Renteria, Hunter S. Waldman<br>*Nutrition Research – Published the 2th December 2019.*						
	**Characteristics of the population**	**Study design**	**Main outcomes**	**Main results**	**Strength and limitations**	**Level of evidence**
**R19**	22 physically active college-age men<br><br>Engaged in 150 min/wk of regular physical activity<br><br>Mean age : 22 ± 2.5 years<br><br>Mean BMI : 28,5 ± 8.3 kg/m²	A 4-week two arm randomized study<br><br>San Marcos, Texas, United States of America<br><br>**Protocol**<br>TRF 8/16 ad libitum (eating to satiation) eveyrday (n=12)<br>Vs<br>TRF 8/16 isocaloric (<± 300kcal difference from baseline)<br>Everyday (n=10)	**Adherence (Caloric time)**<br>Recorded of sheets each day<br><br>**Tolerance** (perceived hunger, satiety, mood, energy, focus and altertness) by VAS every 7 days<br><br>**Caloric intake**<br>Via a net food counter (MyfitnessPal)<br><br>Anthropometric measures and body composition by plethysmgraphy and 7-skinfold methods<br>Restring RH, BP<br><br>**Biomarkers**<br>-lipids<br>-corisol<br>-CRP<br>-adiponectin<br>-IGH<br>-insulin<br>-gluthatione levels<br>-superoxide dimutase (SOD)<br>-nitrate/nitrite (NOx)	**Adherence**<br>Average eating time was 7.2 ± 0.7 hours<br><br>**Tolerance**<br>Higher perceived altertness in ad libitum group<br>Higher perceived energy in ad libitum group<br>Higher perceived focus in ad libitum group<br>Higher perceibed mood in ad libitum group<br><br>**Caloric intake**<br>No change in total caloric intake (p=0.37)<br>Trend ↓ of 22% in fat intake in both groups (p=0.07)<br><br>**Risk factors for CVD**<br>↓ body mass in both groups (p<0.001)<br>↓ body fat mass in both groups (p=0.007) and body fat %<br>↓ systolic blood pressure in both groups<br><br>↑ HDLc in both groups (p=0.005)<br><br>**Biomarkers**<br>↑ adiponectin in both groups (p=0.02)	**Limitation**<br>Groups were different in several variables at the baseline period<br><br>Only three day food log to record baseline data and define isocaloric intake based on these data<br><br>No monitorage of physical activiy : Potiential confusion factor<br><br>Small sample size and short intervention duration : lack of power<br><br>Lack of non-TRF control group<br><br>Healthy active men : limits generalizability of the results<br><br>**Strength**<br>Demonstration of improvements in an already healthy population	HAS : 2<br><br>Validity score : 23/25

**Time-Restricted Eating Effects on Body Composition and Metabolic Measures in Humans with Overweight : A Feasibility Study**<br>Lisa S. Chow, Emily N. C. Manoogian, Alison Alvear Jason G. Fleischer, Honoree Thor, Katrina Dietsche, Qi Wang, James S. Hodges, Nicholas Esch, Samar Malaeb, Tasma Harindhanavudhi, K. Sreekumaran Nair, Satchidananda Panda, and Douglas G. Mashek<br>*The Obesity Society ; Published online 9 April 2020.*						
	**Characteristics of the population**	**Study design**	**Main outcomes**	**Main results**	**Strength and limitations**	**Level of evidence**
**R20**	20 overweight adults<br><br>With prolonged eating window (15.4 ± 0.9 h/d)<br><br>Mean age : 45.5 ± 12 y.<br><br>Mean BMI : 34.1 ± 7.5 kg/m²	A 12-week controlled non-randomized study<br><br>Minneapolis, Minnesota, United States of America<br><br>**Protocol**<br>TRE 8/16 ad libitum eveyrday (n=11)<br>Vs<br>Non-TRE group<br>(n=9)	**Follow-up measures**<br>Adherence (food timing) via asmartphone app (mCC)<br><br>CGMS : during the follow-up period<br><br>Physical activity<br>Via actigraph (ActiGraph Link) on the wrist during the follow-up period<br><br>**Preintervention and postinternvetion measures**<br>Biomarkers<br>-lipid profile<br>-TSH<br>-creatinin<br>-OGTT<br>-HbA1c<br>-HOMA-IR estimation<br><br>Metabolic outcomes : BP, anthropometric measures and body composition by DXA	Adherence to logging on : 83.1%<br><br>Physical activity did no change<br><br>Body weight loss, visceral fat loss and lea mass loss in TRE group compared to non-TRE group<br><br>Body weight loss, fat mass, lean mass and viscrak fat compared to baseline<br><br>Metabolic outcomes : unchanged	**Limitation**<br>Non-randomized trial<br><br>Small sample size and short intervention duration : lack of power<br><br>Male-only cohort : limits gene<br><br>**Strength**<br>Food timing measured by smartphone application<br><br>Continuous monitoring of glucose (CGSM)<br><br>Objective measure of physical activity	HAS : 2<br><br>Validity score : 23/25

A Delayed Morning and Earlier Evening Time- Restricted Feeding Protocol for Improving Glycemic Control and Dietary Adherence in Men with Overweight/Obesity : A Randomized Controlled Trial<br>Evelyn B. Parr, Brooke L. Devlin, Bridget E. Radford and John A. Hawley<br>*Nutrients 2020 – Published : 17 February 2020.*						
	**Characteristics of the population**	**Study design**	**Main outcomes**	**Main results**	**Strength and limitations**	**Level of evidence**
**R21**	11 overweight-obese and sedentary men<br><br>With prolonged eating window (15 h/d)<br><br>Mean age : 38 ± 5 y.<br><br>Mean BMI : 32,2 ± 2.0 kg/m²	A 5-day randomized iso-caloric crossover trial with 10-day wash out period.<br><br>Melbourne, Australia<br><br>**Protocol**<br>TRF 8/16 eveyrday (n=6)<br>Vs<br>Non-TRE group<br>Everyday (n=5)	**Adherence (Food timing)**<br>Recorded of sheets each day<br><br>Body composition DXA<br>REE<br><br>Glycemic metabolism :<br>-CGMS<br>-blood glucose<br>-blood insulin<br><br>Physical activity<br>Via actigraphy (ActiGraph)<br><br>**Tolerance**<br>Dietary compliance, sleep/wake times and activity in diary<br>Semi-structured interviews on subjective perception of the protocole	**Adherence**<br>100% adherence<br><br>**Glycemic metabolism**<br>↓ nocturnal glucose AUC TRE group<br>↓ peak insulin concentrations at breakfast in TRE group<br>↓ peak glucose concentration at breakfast in TRE group<br><br>**Tolerance and perception**<br>Improvement of subjective feelings (well-being and satisfaction)<br>↓ evening hunger in TRE group<br><br>Physical activity unchanged	**Limitation**<br>Small sample size and short intervention duration : lack of power<br><br>Male-only cohort : limits the generalizability of results<br><br>**Strength**<br>Crossover randomized trial<br><br>Iso-caloric protocol<br><br>Objective measure of physical activity	HAS : 2<br><br>Validity score : 21/25

Time-restricted Time-Eating as a Nutrition Strategy for Individuals with Type 2 Diabetes : A Feasibility Study<br>Evelyn B. Parr, Brooke L. Devlin, Karen H. C. Lim, Laura N. Z. Moresi, Claudia Geils, Leah Brennan and John A. Hawley<br>*Nutrients 2020 – Published : 22 October 2020.*						
	**Characteristics of the population**	**Study design**	**Main outcomes**	**Main results**	**Strength and limitations**	**Level of evidence**
**R22**	19-obese adults with type 2 diabetes (HbA1c > 6,5 to <9%)<br><br>Daily eating window > 12h/d<br><br>Mean age : 50 ± 9 years<br><br>Mean BMI : 34 ± 5 kg/m²	A 4-week single-arm non-randomized trial.<br><br>West Wodonga, Australia<br><br>**Protocol**<br>2-week baseline period<br>4-week TRF 9/15 every day.	Body composition DXA, REE, BP<br><br>**Calorie intake**<br>Self-monitored by participants via paper handbook or smartphone app associated with photos of each eating/drinking occasion<br><br>**Glycemic control**<br>-glucose concentrations<br>-insulin concentrations<br><br>**Psychological well-being**<br>-DASS<br>-PSQI<br><br>**Cognitive testing**<br>-CBB	Compliance : 72 ± 24 % of 28 days (i.e., 5 days/week)<br><br>Calorie intake unchanged (adherence to TRE reduced daily energy intake)<br><br>Glycemic control : no significant improvement<br><br>Psychological and cognitive impact : no significant change	**Limitation**<br>Non controlled study<br><br>No monitorage of physical activiy : Potiential confusion factor<br><br>Small sample size and short intervention duration : lack of power<br><br>Adults with type 2 diabetes : limits generalizability of the results	HAS : 4<br><br>Validity score : 20/25

Time-Restricted Eating on Weight Loss and Other Metabolic Parameters in Women and Men With Overweight and Obesity

The TREAT Randomized Clinical Trial

Dylan A. Lowe, PhD ; Nancy Wu, MS; Linnea Rohdin-Bibby, BA; A. Holliston Moore, PhD; Nisa Kelly, MS; Yong En Liu, BS; Errol Philip, PhD; Eric Vittinghoff, PhD; Steven B. Heymsfield, MD; Jeffrey E. Olgin, MD; John A. Shepherd, PhD; Ethan J. Weiss, MD

JAMA Intern Med - Published online September 28, 2020.

	Characteristics of the population	Study design	Main outcomes	Main results	Strength and limitations	Level of evidence
**R23**	105 overweight/obese adults<br><br>Mean age : 46.5 ± 10.5 years	A 12-week two arm randomized study<br><br>San Francisco, California, United States of America<br><br>**Protocol**<br>dTRF 8/16 eveyrday vs control group	**Adherence and sleep quality**<br>Self-reported adherence via surveys through the study app<br><br>**Anthropometric measurement**<br>Weight via a Bluetooth scale provided to each participant<br>Body exposition DXA<br><br>**Biomarkers**<br>-blood lipids<br>-glucose<br>-insuline	**Adherence**<br>83.50% in TRE group<br><br>**Weight**<br>↓ body weight in TRE group (1.17%) compared to baseline that was not significantly different from control group (0.75%).<br>No significant changes in body composition<br><br>**Biomarkers**<br>No significant changes<br><br>**Sleep quality**<br>No significant changes in sleep measures	**Strength**<br>Controlled randomized trial<br><br>Large-size sample<br><br>**Limitation**<br>No monitorage of physical activiy : Potiential confusion factor	HAS : 2<br><br>Validity score : 25/25

Abbreviations : AUC= Aera Under the Curve ; BP= Blood Pressure ; BMI= Body Mass Index ; CBB= Cogstate Brief Battery ; CBC= Cell Blood Count ; CGMS= Continuous Glucose Monitoring System; CVD= Cardiovascular Disease ; DASS= Depression Anxiety Stress Scales ; DXA= Dual-energy X-ray Absorptiometry ; HAS= level of edifence gradation of the High French Authority ; HDLc= High Density Lipoprotein Cholesterol ; HMB= Hydroxy-methyl-butyrate ; HOMA-IR= Homeostasis Accessment of insuline resistance ; LDLc= Low Density Lipoprotein Cholesterol ; mCC= My Circadian Clock ; MetS= Metabolic Syndrom ; OGTT= Oral Glucose Tolerance Test ; PSQI= Pittsburgh Sleep Quality Index ; REE= Resting Energy Expenditure ; RM= Resistance maximal ; RT= Resistance Training ; TRF= Time-restricted feeding; TRE= Time-restricted eating ; dTRF= delayed TRF ; eTRF= earlyTRF; VAS= Visual Analog Scale ; WC = Waist circumference ; WHtR= waist-to height ratio ; y= year(s).

Supplemental Table 2 - Validity score provided by the Downs and Black check-list

Article Author & Name	1	2	3	4	5	6	7	8	9	10	11	12	13	14	15	16	17	18	19	20	21	22	23
**Reporting**																							
1. Is the hypothesis/aim/objective of th e study clearly described ?	1	1	1	1	1	1	1	1	1	1	1	1	1	1	1	1	1	1	1	1	1	1	1
2. Are the main outcomes to be measur ed clearly described in the introduction or Methods section ?<br><br>If the main outcomes are first mentione d in the Results section, the question should be answered no.	1	1	1	1	1	1	1	1	1	1	1	1	1	1	1	1	1	1	1	1	1	1	1
3. Are the characteristics of the patients included in the study clearly described ?<br>In cohort studies and trials, inclusion an d/or exclusion criteria should be given. I n case-control studies, a case-definition and the source for controls sh ould be given.	1	1	1	1	1	1	1	1	1	1	1	1	1	1	1	1	1	1	1	1	1	1	1
4. Are the interventions of interest clear ly described ?<br>Treatments and placebo (where relevan t) that are to be compared should be cle arly described.	1	1	0	1	1	1	1	1	1	1	1	1	1	1	1	1	1	1	1	1	1	1	1
5. Are the distributions of principal conf ounders in each group of subjects to be compared clearly described ?<br>A list of principal confounders is provide d	0	1	0	1	1	0	1	1	1	0	1	1	1	1	1	1	1	1	1	1	1	1	1
6. Are the main findings of the study cle arly described ?<br>Simple outcome data (including denomi nators and numerators) should be reported for all major findings so that th e reader can check the major analyses a nd conclusions.<br>(This question does not cover statistical tests which are considered below).	1	1	1	1	1	1	1	1	1	1	1	1	1	1	1	1	1	1	1	1	1	1	1
7. Does the study provide estimates of t he random variability in the data for the main outcomes ?<br>In non normally distributed data the int erquartile range of results should be rep orted. In normally distributed data the s tandard error, standard deviation or con fidence intervals should be reported. If t he distribution of the data is not describ ed, it must be assumed that the estimat es used were appropriate and the questi on should be answered yes.	1	1	1	1	1	1	1	1	1	1	1	1	1	1	1	1	1	1	1	1	1	1	1
8. Have all important adverse events tha t may be a consequence of the intervent ion been reported ?<br>This should be answered yes if the study demonstrates that there was a compre hensive attempt to measure adverse ev ents. (A list of possible adverse events is provided).	1	1	1	1	1	1	1	1	1	1	1	1	1	1	1	1	1	1	1	0	1	1	1
9. Have the characteristics of patients lo st to follow-up been described ?<br>This should be answered yes where ther e were no losses to follow-up or where losses to follow-up were so small that findings would be unaffected by their inclusion. This shoul d be answered no where a study does n ot report the number of patients lost to follow-up.	1	1	1	1	1	1	1	1	1	1	1	1	1	1	1	1	1	1	1	1	1	1	1
10. Have actual probability values been reported (e.g. 0.035 rather than <0,05)	1	1	1	1	1	1	1	1	1	1	1	1	1	1	1	1	1	1	1	1	1		

for the main outcomes except where the probability value is less than 0,001 ?																							
**External Validity**<br>All the following criteria attempt to addr ess the representativeness of the findings of the study and whether they may be generalised to the population fr om which the study subjects were deriv ed.																					1	1	1
11. Were the subjects asked to participa te in the study representative of the ent ire population from which they were rec ruited ?<br>The study must identify the source popu lation for patients and describe how the patients were selected. Patients would be representative if they comprised the entire source population, an unselected sample of<br>consecutive patients, or a random samp le. Random sampling is only feasible wh ere a list of all members of the relevant population exists. Where a study does n ot report the proportion of the source p opulation from which the<br>patients are derived, the question shoul d be answered as unable to determine.	0	1	0	1	1	1	0	0	0	0	0	0	0	0	0	0	0	0	0	0	0	0	1
12. Were those subjects who were prep ared to participate representative of the entire population from which they were recruited ?<br>The proportion of those asked who agre ed should be stated. Validation that the sample was representative would includ e demonstrating that the distribution of the main confounding factors was the sa me in the study sample and the source population.	0	1	0	0	0	1	1	0	1	0	0	0	1	1	1	0	0	0	0	1	0	0	1
13. Were the staff, places, and facilities where the patients were treated, repres entative of the treatment the majority o f patients receive ?<br>For the question to be answered yes the study should demonstrate that the inte rvention was representative of that in u se in the source population. The questio n should be answered no if, for example , the intervention was<br>undertaken in a specialist centre unrepr esentative of the hospitals most of the s ource population would attend.	1	1	1	1	1	1	1	1	1	1	1	0	1	1	1	1	1	1	1	1	1	1	1
**Internal validity – bias**																							
14. Was an attempt made to blind study subjects to the intervention they have r eceived ?<br>For studies where the patients would ha ve no way of knowing which interventio n they received, this should be answere d yes.																							
15. Was an attempt made to blind those measuring the main outcomes of the in tervention ?																							
16. If any of the results of the study wer e based on « data dredging », was this made clear ?<br>Any analyses that had not been planned at the outset of the study should be cle arly indicated. If no retrospective unpla nned subgroup analyses were reported, then answer yes.	1	1	1	1	1	1	1	1	1	1	1	1	1	1	1	1	1	1	1	1	1	1	1
17. In trials and cohort studies, do the a nalyses adjust for different lengths of fol low-up of patients, or in case-<br>control studies, is the time period betwe	1	X	1	0	X	1	0	1	0	1	1	1	X	1	1	1	1	1	1	1	1	1	1

en the intervention and outcome the same for cases and controls ? Where follow-up was the same for all study patients the answer should yes. If different lengths of follow-up were adjusted for by, for example, survival analysis the answer should be yes. Studies where differences in follow-up are ignored should be answered no.																							
18. Were the statistical tests used to assess the main outcomes appropriate ? The statistical techniques used must be appropriate to the data. For example nonparametric methods should be used for small sample sizes. Where little statistical analysis has been undertaken but where there is no evidence of bias, the question should be answered yes. If the distribution of the data (normal or not) is not described it must be assumed that the estimates used were appropriate and the question should be answered yes.	1	1	1	1	1	1	1	1	1	1	1	1	1	1	1	1	1	1	1	1	1	1	1
19. Was compliance with the intervention/s reliable ? Where there was noncompliance with the allocated treatment or where there was contamination of one group, the question should be answered no. For studies where the effect of any misclassification was likely to bias any association to the null, the question should be answered yes.		1	1	1	1	1	1	1	1	1	1	0	1	1	1	1	1	1	1	1	1	1	1
20. Were the main outcome measures used accurate (valid and reliable) ? For studies where the outcome measures are clearly described, the question should be answered yes. For studies which refer to other work or that demonstrates the outcome measures are accurate, the question should be answered as yes.	1	1	1	1	1	1	1	1	1	1	1	1	1	1	1	1	1	1	1	1	1	1	1
**Internal validity - confounding (selection bias)**																							
21. Were the patients in different intervention groups (trials and cohort studies) or were the cases and controls (case-control studies) recruited from the same population ? For example, patients for all comparison groups should be selected from the same hospital. The question should be answered unable to determine for cohort and casecontrol studies where there is no information concerning the source of patients included in the study	1	1	1	1	1	1	0	1	0	0	1	1	1	1	1	1	1	1	1	1	1	1	1
22. Were study subjects in different intervention groups (trials and cohort studies) or were the cases and controls (case-control studies) recruited over the same period of time ? For a study which does not specify the time period over which patients were recruited, the question should be answered as unable to determine.	1	1	1	1	1	1	0	1	0	1	1	0	1	1	1	1	1	1	1	1	1	1	1
23. Were study subjects randomised to intervention groups ? Studies which state that subjects were randomized should be answered yes except where method of randomisation would not ensure random allocation. For example alternate allocation would score no because it is predictable	0	1	0	1	1	0	1	1	0	0	1	1	1	1	0	0	0	0	1	0	1	0	1
24. Was the randomised intervention assignment concealed from both patients	0	1	0	1	1	0	0	1	0	0	1	1	1	1	0	0	0	0	1	0	1	0	1

and health care staff until recruitment was complete and irrevocable ? All nonrandomised studies should be answered no. If assignment was No concealed from patients but not from staff, it should be answered no																							
25. Was there adequate adjustment for confounding in the analyses from which the main findings were drawn ? This question should be answered no for trials if : the main conclusions of the study were based on analyses of treatment rather than intention to treat ; the distribution of known confounders in the different treatment groups was not described; or the distribution of known confounders differed between the treatment groups but was not taken into account in the analyses. In nonrandomized studies if the effect of the main confounders was not investigated or confounding was demonstrated but no adjustment was made in the final analyses the question should be answered as no	0	1	0	1	1	0	0	1	1	0	1	1	1	1	1	0	1	1	1	1	1	1	1
26. Were losses of patients to follow-up taken into account ? If the numbers of patients lost to follow-up are not reported, the question should be answered as unable to determine. If the proportion lost to follow-up was too small to affect the main findings, the question should be answered yes.	1	0	1	1	1	1	1	1	1	1	1	1	1	1	1	1	1	1	1	1	1	1	1
**Power**																							
27. Did the study have sufficient power to detect a clinically important effect where the probability value for a difference being due to chance is less than 5% ? Sample sizes have been calculated to detect adifference of x% and y%.	0	1	0	1	0	0	0	1	0	0	1	0	1	0	0	☒	1	1	1	1	0	0	1
**Total score /25**	17	23	17	23	22	20	18	23	18	17	23	19	23	23	21	19	21	21	23	21	22	20	25

Legend : 1 : Yes/ 0 : No / ☒ Unable to determine

Supplemental Table 3 – HAS gradation

<table>
<tr><th>level of scientific evidence provided by the literature</th><th>Grade of guidelines</th></tr>
<tr><td>Level 1
High power randomized controlled trials
Meta-analyze of randomized controlled trials
Decision analysis based on well-conducted trials</td><td>A
Established scientific evidence</td></tr>
<tr><td>Level 2
Low power randomized controlled trials
Non-randomized controlled well-conducted trials
Cohort study</td><td>B
Scientific presumption</td></tr>
<tr><td>Level 3
Case control trials</td><td rowspan="2">C
Low level of scientific evidence</td></tr>
<tr><td>Level 4
Controlled trials with major biases
Retrospective trials
Cases series
Descriptive epidemiological studies</td></tr>
</table>

Level of evidence provided by the High French Health Autorithy (HAS).

Supplemental Method 1 - Research equations

Pubmed

"time-restricted feeding"[All Fields] OR "time-restricted eating"[All Fields] OR (time-restricted[All Fields] AND ("nutritional status"[MeSH Terms] OR ("nutritional"[All Fields] AND "status"[All Fields]) OR "nutritional status"[All Fields] OR "nutrition"[All Fields] OR "nutritional sciences"[MeSH Terms] OR ("nutritional"[All Fields] AND "sciences"[All Fields]) OR "nutritional sciences"[All Fields])) AND ("2014/01/01"[PDat] : "2020/09/29"[PDat] AND "humans"[MeSH Terms])

Web of Science

TS=(« time-restricted feeding » OR « time-restricted eating » OR « time-restricted nutrition »)

Indexes=SCI-EXPANDED, SSCI, A&HCI, CPCI-S, CPCI-SSH, BKCI-S, BKCI-SSH, ESCI Timespan=Last 6 years

Scopus

TITLE-ABS-KEY ("time-restricted feeding") OR TITLE-ABS-KEY ("time-restricted eating") OR TITLE-ABS-KEY ("time-restricted nutrition") AND (LIMIT-TO (PUBYEAR , 2020) OR LIMIT-TO (PUBYEAR , 2019) OR LIMIT-TO (PUBYEAR , 2018) OR LIMIT-TO (PUBYEAR , 2017) OR LIMIT-TO (PUBYEAR , 2016) OR LIMIT-TO (PUBYEAR , 2015) OR LIMIT-TO (PUBYEAR , 2014))

Subject area : Medicine

Science Direct

Year: 2014-2020 Title, abstract, keywords: "time-restricted feeding" OR "time-restricted eating" OR "time-restricted nutrition"

Cochrane Library

Year: 2014-2020

"time-restricted feeding" in Title Abstract Keyword OR "time-restricted eating" in Title Abstract Keyword OR "time-restricted nutrition" in Title Abstract Keyword - (Word variations have been searched)

Nutrition Reviews – Oxford Academic

"time-restricted feeding" OR "time-restricted eating" OR "time-restricted nutrition"

Published: January 2014 to September 2020

Nutrition Reviews - Wiley Online Library

""time restricted feeding"" in Title

01/2014 à 09/2020

Obesity – Wiley Online Library

"time-restricted feeding" OR "time-restricted eating" OR "time-restricted nutrition"

Published: January 2014 to September 2020

American journal of clinical nutrition

"time-restricted feeding" OR "time-restricted eating" OR "time-restricted nutrition"

Published: January 2014 to September 2020

Nutrition - Annual Review of Nutrition

"time-restricted feeding" OR "time-restricted eating" OR "time-restricted nutrition"

Last 6 years

Clinical nutrition

You searched for "time-restricted feeding" in All Content OR "time-restricted eating" in All Content OR "time-restricted nutrition"

6 last years
